# Phytochemical Profile and Antioxidant Activity of the Tuber and Peel of *Pachyrhizus erosus*

**DOI:** 10.3390/antiox14040416

**Published:** 2025-03-31

**Authors:** Jing Xiang, Shiting Huang, Xingyu Wu, Yixuan He, Haiyan Shen, Shuangyang Tang, Fengyuan Zhu, Ying Luo

**Affiliations:** Institute of Biochemistry and Molecular Biology, Hunan Provincial Key Laboratory for Special Pathogens Prevention and Control, Hengyang Medical College, University of South China, Hengyang 421001, China; xiangj666@163.com (J.X.); shea1119@163.com (S.H.); wuxingyu212@126.com (X.W.); 16673565535@163.com (Y.H.); 2015002076@usc.edu.cn (H.S.); tsy7909@126.com (S.T.); 19196136980@163.com (F.Z.)

**Keywords:** *Pachyrhizus erosus*, chemical composition, antioxidant activity, UPLC-Orbitrap-MS/MS

## Abstract

This study systematically investigated the antioxidant activities and phytochemical profiles of petroleum ether (PE), ethyl acetate (EtOAc), n-butanol (n-BuOH) and aqueous fractions of edible (tubers) and non-edible portions (peels) of *Pachyrhizus erosus.* The results showed that both the tubers and peels from *P. erosus* were rich in polyphenols and flavonoids, whereas the EtOAc fraction of peels had the highest polyphenol content, and the PE fraction of peels had the highest total flavonoid content. ABTS, DPPH, and FRAP assays revealed that both the EtOAc fraction of tubers and peels from *P. erosus* showed significant antioxidant activity, whereas the EtOAc fraction of peels possessed better antioxidant activity than that of tubers. UPLC-Orbitrap-MS/MS analysis indicated that thirty compounds were identified from the EtOAc fractions of peels and tubers, including twenty-one flavonoids, six phenolics, two coumarins, and one lignan, some of which have previously been revealed to display significant antioxidant and anti-inflammatory effects via the Nrf2-Keap1 and NF-κB signaling pathways. These findings provide robust scientific evidence for the health-promoting properties and pharmaceutical potential of *P. erosus*, and its non-edible portion (peels) has great potential for use as a natural antioxidant in the food, cosmetic, and pharmaceutical industries.

## 1. Introduction

Oxidative stress is caused by excessive free radicals, which play a crucial role in the pathogenesis of various chronic diseases, such as cardiovascular diseases, atherosclerosis, metabolic syndrome, diabetes, neurodegenerative disorders, and cancer [1]. Many antioxidants commonly exist in fruits and vegetables, which can effectively neutralize free radicals, inhibit oxidative enzymes, and chelate metals, thereby preventing oxidative stress-related damage [2].

In recent years, increasingly more attention has been paid to the health-promoting effects of natural antioxidants. Numerous epidemiological studies and clinical trials have demonstrated that diets rich in natural antioxidants can protect the human body from oxidative stress and reduce the incidence of chronic diseases (e.g., cardiovascular diseases, cancer) [3]. Therefore, enhancing the intake of foods containing high natural antioxidants is vital for maintaining overall health and reducing the risk of chronic diseases.

*Pachyrhizus erosus*, a perennial plant from the Fabaceae family, is also known as bang kuang (China), jicama (Mexico), Kuzu-imo (Japan), Sankalu (India), pupur dingin (Indonesia), and yam bean (the United States) [4]. Native to Mexico and Central America, *P. erosus* is now widely cultivated in tropical and sub-tropical regions globally, including China, the United States, Brazil, Philippines, Indonesia, Thailand, India, and some African countries. The tubers of *P. erosus* are rich in various nutrients, proteins, carbohydrates, polysaccharides (inulin), vitamins, and minerals, and are low in calories and abundant in dietary fiber, making them one of the top ten health foods globally [5]. In addition, the tubers of *P. erosus* have also been traditionally utilized for medical applications due to their diverse pharmacological activities [6].

Previous phytochemical studies have reported about 50 compounds that have been isolated and identified mainly from the seeds, tubers, and leaves of *P. erosus*, including flavonoids, triterpenes, phenolics, saponins, organic acids, and fatty acids, which contribute to the diverse biological activities of *P. erosus*, such as antioxidant, anti-aging, anti-cancer, anti-diabetes, immunomodulatory, antiviral, antiosteoporosis, skin whitening, gastroprotective, and anti-obesity effects [6].

Early studies demonstrated that the tubers of *P. erosus* exhibited good antioxidant properties. For instance, Choi revealed that the ethyl acetate extract from the tubers from *P. erosus* exhibited the highest antioxidant activity in both DPPH and ABTS radical scavenging assays [5]; Bhanja found that the inulin in the tubers of *P. erosus* showed good antioxidant and in vitro probiotic effects, indicating its role in promoting gut health [7]; Laovachirasuwan indicated that the dichloromethane extract from the tubers from *P. erosus* exhibited significant anti-melanogenesis and antioxidant effects, positioning it as a promising source of natural antioxidants and anti-melanogenesis agents for use in cosmetic products [8]. Additionally, regarding the antioxidant activity of peels from *P. erosus*, only Siregar reported that the 70% ethanol extract of peels presented strong DPPH scavenging activity [9]. Despite several studies reporting on the antioxidant activities of tubers and peels from *P. erosus*, systematic evaluations of their antioxidant effects on a chemical basis have not been fully elucidated.

Thus, this study focused on comparing the total polyphenol content (TPC), total flavonoid content (TFC), and antioxidant activities (DPPH, ABTS, and FRAP assays) of petroleum ether (PE), ethyl acetate (EtOAc), n-butanol (n-BuOH), and aqueous fractions of tubers and peels from *P. erosus*, as well as on analyzing the chemical compositions of their strongly active fraction using UPLC-Orbitrap-MS/MS. The goal of this study was to elucidate the chemical basis of the antioxidant activity of *P. erosus* and further explore the potential utilization of the peels of *P. erosus* which are often discarded. This research will provide a theoretical basis for the application of the peels and tubers of *P. erosus* as a natural antioxidant in the food, cosmetic, and pharmaceutical industries.

## 2. Materials and Methods

### 2.1. Chemicals and Reagents

Folin–Ciocalteu reagent was acquired from Macklin (Shanghai, China). 2,2-diphenyl-1-picrylhydrazyl (DPPH), 2,4,6-tris-(2-pyridyl)-s-triazine (TPTZ), 2,2′-azino-bis (3-ethylbenzothiazoline-6-sulfonic acid) (ABTS), rutin, and gallic acid were obtained from Aladdin Biotechnology Co., Ltd. (Shanghai, China). LC- and MS-grade methanol, acetonitrile, formic acid, and isopropanol were purchased from ANPEL (Shanghai, China). Analytical grade ethanol, methanol, petroleum ether, ethyl acetate, and n-butanol were purchased from Sinopharm Chemical Reagent Co., Ltd. (Beijing, China). Ultrapure water was prepared using a Milli-Q water purification system (Millipore, Bedford, MA, USA).

### 2.2. Plant Materials and Extract Preparation

*P. erosus* was collected from Loudi City, Hunan Province. After washing, the peels and tubers of *P. erosus* were manually separated. The dried peels and tubers (500 g) were then subjected to extraction with 95% ethanol at room temperature, respectively. Subsequently, the samples were rotary evaporated and concentrated to afford the ethanol extracts. The resulting ethanol extracts were suspended in H_2_O and successively partitioned with petroleum ether, ethyl acetate, and n-butanol to afford a petroleum ether fraction (PE), ethyl acetate fraction (EtOAc), n-butanol fraction (n-BuOH), and aqueous fraction (Aqueous) after condensation to dry material in vacuo. The yields of the four fractions (PE, EtOAc, n-BuOH, and Aqueous) from *P. erosus* peels were 3.158 g, 3.993 g, 5.521 g, and 47.761 g, respectively. Conversely, the yields of the four fractions (PE, EtOAc, n-BuOH, and Aqueous) from *P. erosus* tubers were 1.101 g, 0.923 g, 14.892 g, and 102.057 g, respectively.

### 2.3. Antioxidant Activity Assays

#### 2.3.1. ABTS Radical Scavenging Activity

The ABTS radical scavenging activity was determined using a previously described method [10] with slight modifications. First, we mixed 5 mL ABTS (7.0 mmol/L) with 88 µL K_2_S_2_O_8_ (140 mmol/L) and let the mixture stand in the dark at room temperature for 12–16 h to prepare ABTS working solution. The ABTS solution was diluted with 10 mmol/L phosphate-buffered saline (PBS) to adjust the absorption value to 0.70 ± 0.02 at 734 nm. Then, 200 µL of the ABTS working solution was mixed with 10 µL of the sample in a 96-well microplate. Then, the mixture was reacted for 6 min. The absorbance of the mixture was measured at 405 nm. The ABTS radical scavenging activity was calculated using the following formula: ABTS radical scavenging rate (%) = [1 − (A_1_ − A_2_)/A_0_] × 100%, where A_0_ is the absorbance of ABTS and DMSO; A_1_ is the absorbance with the sample reaction solution; and A_2_ is the absorbance of sample and PBS.

#### 2.3.2. DPPH Radical Scavenging Activity

The DPPH radical scavenging activity was performed using the previous method [11] with slight modifications. Briefly, 20 µL of the sample was mixed with 180 µL of 0.1 mM DPPH methanol solution. After incubation in the dark at room temperature for 30 min, the absorbance was measured at 515 nm using a microplate reader. The DPPH radical scavenging activity was calculated using the following formula: DPPH radical scavenging rate (%) = [1 − (A_1_ − A_2_)/A_0_] × 100%, where A_0_ is the absorbance of the DPPH without samples, A_1_ is the absorbance of samples with DPPH, and A_2_ is the absorbance of samples without DPPH.

#### 2.3.3. Ferric Reducing Antioxidant Power (FRAP) Assay

The FRAP assay was assessed according to the previously method [12] with some modifications. FRAP working solution was prepared with 0.3 M acetate buffer (pH 3.6), and 10 mM 2,4,6-tris-(2-pyridyl)-s-triazine (TPTZ) in 40 mM HCl solution, and 20 mM FeCl_3_ solution in a 10:1:1 (*v*/*v*/*v*) ratio. 20 µL of sample and 180 µL of FRAP working solution were mixed in a 96-well plate and incubated at 37 °C for 30 min in the dark. The absorbance of the mixture was measured at 593 nm. Ferrous sulfate (FeSO_4_) solutions (0.6–2.1 mM) were used to construct the standard curve in the same way. The FRAP value of the samples were calculated based on the linear standard curve and expressed as mmol FeSO_4_ equivalents per gram of sample (mmol/g FeSO_4_ equivalents).

### 2.4. Determination of Total Polyphenol and Flavonoid Content

#### 2.4.1. Total Polyphenol Content

The total polyphenol content was determined using the Folin–Ciocalteu method [13] with some modifications. Briefly, 0.1 mL of extract (1 mg/mL) and 0.1 mL of Folin–Ciocalteu reagent (1 N) were mixed and reacted with 2.8 mL of distilled water in a 10 mL brown volumetric flask for 8 min. Then, 2 mL of 7.5% Na_2_CO_3_ solution was added and incubated in a water bath at 37 °C for 1 h. The absorbance of the mixture was measured at 765 nm. Gallic acid (0–0.3 mg/mL) was used as the standard to establish the calibration curve. The total polyphenol content of the extracts was expressed as mg of gallic acid equivalents (GAE)/g extract.

#### 2.4.2. Total Flavonoid Content

The total flavonoid content was determined using the aluminum chloride colorimetric method [14] with some modifications. Briefly, 1.0 mL of extract (1 mg/mL) and 0.3 mL of 5% NaNO_2_ solution were mixed and reacted with 4.0 mL of distilled water in a 10 mL brown volumetric flask for 5 min. Then, 0.3 mL of 10% AlCl_3_ solution was added and retained in the dark for 6 min. Afterward, 2.0 mL of 1.0 M NaOH solution was added, and then the final volume was made up to 10 mL with distilled water. The absorbance of the mixture was measured at 510 nm. Rutin (0–0.5 mg/mL) was used as the standard to establish the calibration curve. The total flavonoid content of the extracts was expressed as mg of rutin equivalents (RE)/g extract.

### 2.5. UPLC-Orbitrap-MS/MS Analysis

The ethyl acetate fractions of tubers and peels from *P. erosus* (10 mg) was dissolved in 10 mL methanol, respectively, then filtered through a 0.22 μm membrane and stored at −20 °C.

The qualitative determination of chemical composition of the ethyl acetate fractions of tubers and peels from *P. erosus* was conducted using the UPLC-Orbitrap-MS system (UPLC, Vanquish; MS, HFX). Separation was performed on a Waters HSS T3 column (100 mm × 2.1 mm, 1.8 μm) (Waters Corporation, Milford, MA, USA). The column temperature was 40 °C. The mobile phase A was Milli-Q water with 0.1% formic acid and B was acetonitrile with 0.1% formic acid. The gradient program was performed as follows: 0 min, A/B (100:0, *v*/*v*); 1 min, A/B (100:0, *v*/*v*); 12 min, A/B (5:95, *v*/*v*); 13 min, A/B (5:95, *v*/*v*); 13.1 min, A/B (100:0, *v*/*v*); 17 min, A/B (100:0, *v*/*v*). The injection volume was set to 2.0 μL, and the flow rate was set at 0.3 mL/min.

HRMS data were recorded on a Q Exactive HFX Hybrid Quadrupole Orbitrap mass spectrometer equipped with a heated ESI source (Thermo Fisher Scientific, Waltham, MA, USA) utilizing the Full-ms-ddMS2 acquisition methods. The ESI source parameters were set as follows: sheath gas pressure, 40 arb; aux gas pressure, 10 arb; spray voltage, +3000 V/−2800 V; temperature, 350 °C; and ion transport tube temperature, 320 °C. The scanning range of the primary mass spectrometry (*m*/*z* range) was 70–1050 Da, with a primary resolution of 70,000 and secondary resolution of 17,500. Thermo scientific Xcalibur 4.1 software was applied to acquire and deal with the original data.

### 2.6. Statistical Analysis

Experimental data are expressed as the mean ± standard deviation (SD) of three independent experiments. Statistical analysis of experimental data by *t*-test or one-way analysis of variance (ANOVA) was performed using GraphPad Prism 9.5.1 software (San Diego, CA, USA), with *p* ≤ 0.05 considered significant.

## 3. Results

### 3.1. Antioxidant Activity

Oxidative stress is associated with many chronic diseases and aging [15]. Antioxidants can react differently with various free radicals or oxidizing agents that are crucial for protecting biological systems from oxidative stress. Generally, one method is not sufficient for predicting the antioxidant activity of plant extracts; thus, it is necessary to use multiple methods, such as DPPH, ABTS radical scavenging and ferric reducing antioxidant power (FRAP) to comprehensively evaluate the antioxidant activity [16,17].

#### 3.1.1. ABTS Radical Scavenging Activity

**Figure 1:**
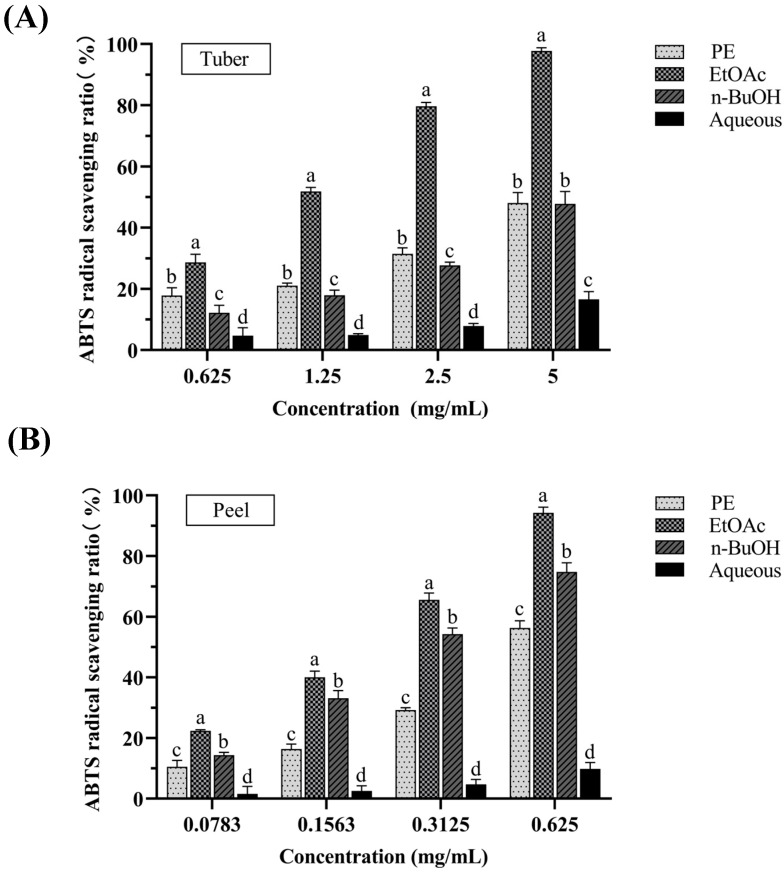
The ABTS radical scavenging activities of tuber (**A**) and peel (**B**) extracts from *P. erosus*. Results are expressed as mean ± SD (n = 3). Different letters as superscript were significantly different (*p* < 0.05).

The ABTS radical scavenging activities of PE, EtOAc, n-BuOH, and aqueous fractions of tubers and peels from *P. erosus* are shown in Figure 1A,B. As depicted, the inhibition of the ABTS radical scavenging activity of each fraction exhibited a concentration-dependent trend. Among the fractions from the tubers of *P. erosus*, the EtOAc fraction possessed the greatest ABTS radical scavenging capacity with an IC_50_ value of 1.126 ± 0.089 mg/mL. Similarly, among the fractions from the peels of *P. erosus*, the EtOAc fraction also presented the greatest ABTS radical scavenging capacity with an IC_50_ value of 0.1749 ± 0.017 mg/mL. Comparing the tuber and peel extracts, both peels and tubers showed some ABTS radical scavenging capacity. Furthermore, the ABTS radical scavenging activities of PE, EtOAc, n-BuOH, and the aqueous fractions of peels were noticeably higher than that of the tubers. This trend was especially evident in the EtOAc fraction at a dose of 0.625 mg/mL, which demonstrated that the EtOAc fractions from the peels of *P. erosus* displayed the most outstanding antioxidant activity.

#### 3.1.2. DPPH Radical Scavenging Activity

**Figure 2:**
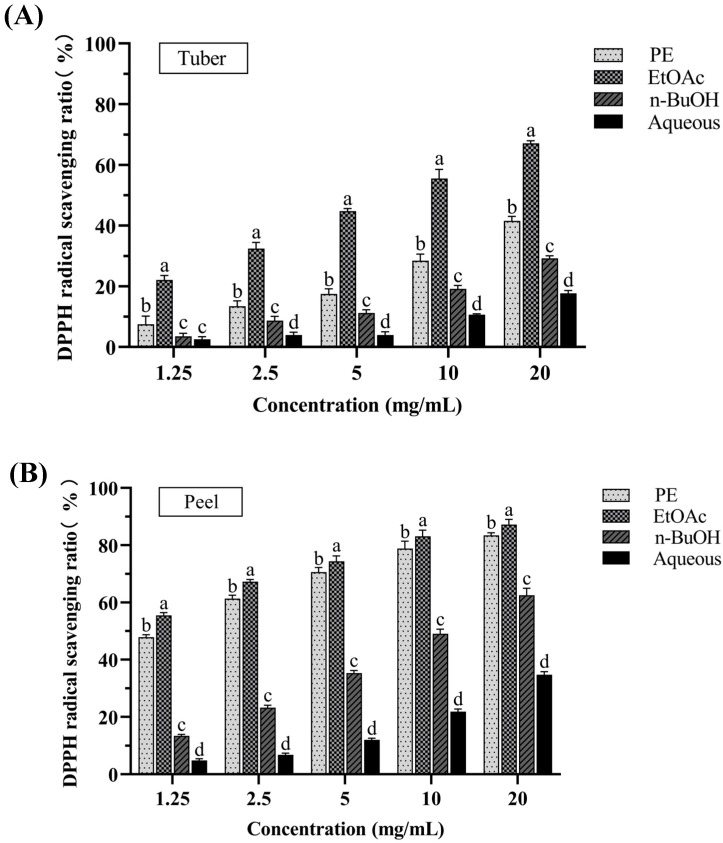
The DPPH radical scavenging activities of tuber (**A**) and peel (**B**) extracts from *P. erosus*. Results are expressed as mean ± SD (n = 3). Results with different letters as superscript were significantly different (*p* < 0.05).

The DPPH radical scavenging assay is a common method used for evaluating the antioxidant capacity of plant extracts by assessing the reduction in DPPH radicals [18]. Figure 2A,B shows the DPPH radical scavenging activities of PE, EtOAc, n-BuOH, and aqueous fractions of tubers and peels from *P. erosus* at different concentrations (1.25, 2.5, 5, 10, 20 mg/mL). For all the extracts, the inhibition of the DPPH activity was concentration dependent. Among the fractions of tubers, the EtOAc fraction exhibited the greatest DPPH radical scavenging capacity with an IC_50_ value of 6.443 ± 0.595 mg/mL. Similarly, the EtOAc fraction of peels also presented the greatest DPPH radical scavenging capacity with an IC_50_ value of 0.772 ± 0.078 mg/mL. Comparing the tuber and peel extracts, both peels and tubers showed good scavenging effects on DPPH radicals. Particularly in the EtOAc fraction, the antioxidant activity of the peels was significantly higher than that of the tubers.

#### 3.1.3. FRAP Assay

The FRAP (ferric-reducing antioxidant power) assay is a widely used colorimetric method for evaluating the antioxidant effects of various plant extracts [19]. Specifically, this method measures the ability of antioxidants to reduce the colorless Fe (III)-TPTZ complex to the blue-colored Fe (II)-TPTZ complex. The intensity of the blue color, measured spectrophotometrically at 593 nm, correlates with the antioxidant capacity of the substance. The FRAP values of PE, EtOAc, n-BuOH, and aqueous fractions of tubers and peels from *P. erosus* are shown in Table 1. FeSO_4_ was used to establish a standard curve and its linear equation was y = 0.4196x + 0.0023, R^2^ = 0.9997. Among these fractions, the EtOAc fraction of peels showed the higher FRAP value (12.56 ± 1.720 mM Fe (II)/g extract) than that of the EtOAc fraction of tubers (4.113 ± 1.158 mM Fe (II)/g extract), indicating that the peels exhibited the strongest Fe^3+^-reducing power.

### 3.2. Determination of Total Polyphenol and Flavonoid Content

**Figure 3:**
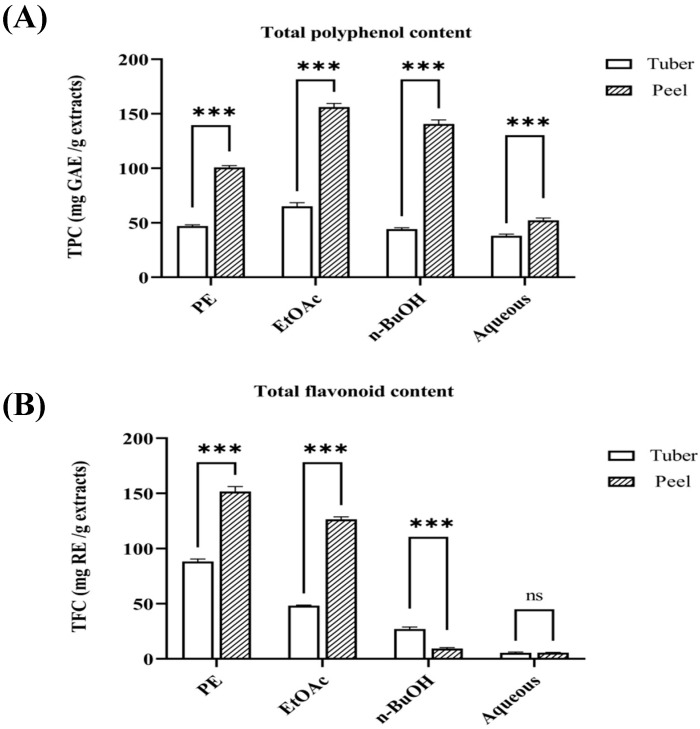
Total polyphenol content (TPC) and total flavonoid content (TFC) of tuber and peel extracts from *P. erosus*. (**A**) Total polyphenol content (TPC) expressed as mg GAE/g extract. (**B**) Total flavonoid content (TFC) expressed as mg RE/g extract. Results are presented as mean ± SD (n = 3). Significant differences between tuber and peel at the same extraction solvent are indicated by asterisks (*** *p* < 0.001, ns: not significant).

Total polyphenol compounds and flavonoids are widely distributed in plants, which are significantly positively correlated with antioxidant activity. For example, plants (sweet chestnut and sweet rowan) contain higher levels of these compounds, which are associated with stronger antioxidant activity [20,21]. Figure 3A,B shows the total polyphenol and flavonoid contents of the PE, EtOAc, n-BuOH, and aqueous fractions of tubers and peels from *P. erosus*. The results indicated that the EtOAc fraction of peels had the highest polyphenol content (156.2 ± 3.282 mg GAE/g extract), while the PE fraction of peels had the highest total flavonoid content (151.7 ± 4.510 mg RE/g extract). Notably, within the same fraction, the total polyphenol content in the peels was significantly higher than that in the tubers (*p* < 0.05). In contrast, the n-BuOH fraction of tuber had higher total flavonoid content than that of peel (*p* < 0.05). The peels of *P. erosus* contain higher amounts of total polyphenol sand total flavonoids, which could be attributed to its prolonged exposure to environmental factors and direct contact with soil, necessitating more polyphenols and flavonoids to counteract environmental stresses and pest attacks. Besides, these high content polyphenol and flavonoids may play an important role in antioxidant activity of peels from *P. erosus* (Supplementary Materials, Figure S32).

### 3.3. Identification of Chemical Constituents of the Strong Active Ethyl Acetate Fractions of Tubers and Peels from P. erosus

To date, several studies have investigated the antioxidant activities of tubers and peels from *P. erosus*, but systematic evaluations on the chemical basis of their antioxidant effect have not yet been fully carried out. In this study, using antioxidant activity screening, it was found that the EtOAc fractions of peels and tubers from *P. erosus* showed the best antioxidant activity. The chemical compositions of the EtOAc fractions of peels and tubers were characterized by UPLC-Orbitrap-MS/MS.

Based on retention time, molecular formula, detected negative or positive ionization modes, mass error (<3 ppm), mass-to-charge ratio, and the MS/MS fragment ions, thirty compounds were identified from the strongly active EtOAc fractions of peels and tubers (Figure 4 and Table 2), including twenty-one flavonoid compounds (ten isoflavones, six flavonoid glycosides, three chalcones, two methoxy flavones), six phenolic compounds, two coumarins, and one lignan. The total ion chromatograms (TIC) in both positive and negative ionization modes and MS/MS2 spectrum of compounds are shown in the Supplementary Materials (Figures S1–S31 and Table S1).

## 4. Discussion

Increasing evidence suggests that oxidative stress and inflammation are key factors in many chronic diseases [22]. Natural antioxidants, such as flavonoids, phenolic compounds, and coumarins, are commonly used in daily life, which can alleviate oxidative stress and inflammatory responses, thus improving the prognosis of these chronic diseases. *P. erosus* belongs to the Fabaceae family, whose tubers are abundant in various nutrients and dietary fiber, and is considered one of the top ten health foods globally [5]. Previous studies demonstrated that the tubers and peels of *P. erosus* possessed antioxidant activities. However, to date, the chemical basis of their antioxidant effect has still not been fully elucidated. Therefore, this study focused on evaluating the total polyphenol and total flavonoid content, antioxidant activities, and phytochemical profile of PE, EtOAc, n-BuOH, and aqueous fractions of tubers and peels from *P. erosu,* which aimed to elucidate the chemical basis of the antioxidant activity of *P. erosus* and further explore the potential utilization of the peels of *P. erosus* which are often discarded.

The results indicated that the EtOAc fraction of peels had the highest polyphenol content, while the PE fraction of peels had the highest total flavonoid content. Though ABTS, DPPH, and FRAP assays, it was found that both the EtOAc fraction of tubers and peels from *P. erosus* showed significant antioxidant activity, whereas the EtOAc fraction of peels possessed better antioxidant activity than that of tubers. These findings are consistent with those documented by Choi et al., who established that the EtOAc fraction of the tuber from *P. erosus* exhibits notable DPPH and ABTS radical scavenging activities [5]. Additionally, Siregar demonstrated that the peel extracts from *P. erosus* displayed stronger DPPH radical scavenging activity than that of tuber extracts [9]. This discovery further supports our present results, which highlights the superior antioxidant activity of the peel from *P. erosus*.

To further elucidate the chemical basis of antioxidant activities, UPLC-Orbitrap-MS/MS was employed to analyze the chemical compositions of the EtOAc fractions obtained from both peels and tubers. A total of thirty compounds were identified, including twenty-one flavonoid compounds (ten isoflavones, six flavonoid glycosides, three chalcones, and two methoxy flavones), six phenolic compounds, two coumarins, and ome lignan. In particular, except for five compounds (genistin, daidzin, daidzein, formononetin, and salicylic acid) that were previously reported in the tubers of *P. erosus* [5,6], the other remaining compounds were found in the tubers of *P. erosus* for the first time. As far as we know, this is the first time that the phytochemical profiles of peels from *P. erosus* have been analyzed and studied. Thirty compounds were identified in the peels, which were mainly flavonoids and phenolics. The identification of these compounds not only contributes to a more detailed understanding of the phytochemical profile of *P. erosus*, but also offers insights into the observed significant antioxidant activity.

Among these compounds, many have been reported to exhibit significant pharmacological activities, including cardioprotective, anti-cancer, antidiabetic, antiepileptic, neuroprotective, gastric ulcer preventive, antidepressant, antiallergic, anti-osteoporosis, antimicrobial, anti-thrombosis, and anticonvulsant effects [23,24,25,26,27,28,29,30,31,32,33,34,35,36,37,38,39,40,41,42,43,44,45,46,47,48,49,50,51,52,53] (Table 3).

Nuclear factor E2-related factor 2 (Nrf2) is a widely present transcription factor, which binds to the antioxidant response element (ARE), and activates a series of antioxidant genes, thereby regulating the expression of various antioxidant enzymes, such as heme oxygenase-1 (HO-1), NAD(P)H quinone dehydrogenase 1 (NQO1), superoxide dismutase (SOD), catalase (CAT), and glutathione peroxidase (GSH-Px). Under homeostasis conditions, Nrf2 is retained in the cytoplasm by binding to its inhibitory protein Keap1. The E3 ubiquitin ligase complex formed by Keap1 and Cullin 3 (CUL3) mediates the ubiquitination and degradation of Nrf2, thus inhibiting its activity. However, under the conditions of oxidative stress, Nrf2 is released from Keap1 and translocated to the nucleus, which activates massive gene expression of antioxidant enzyme to exert an antioxidant effect. This major antioxidant regulatory mechanism has been validated in several of the metabolites we screened. For example, esculetin (**28**) can activate the ERK signaling pathway and promote Nrf2 phosphorylation and nuclear translocation, thereby upregulating NQO1 expression and significantly alleviating H_2_O_2_-induced oxidative damage in C2C12 cells [54]. In a myocardial ischemia–reperfusion injury model, isoliquiritigenin (**19**) reduces oxidative stress and ferroptosis by promoting Nrf2 nuclear translocation and activating the expression of HO-1, SLC7A11, and GPX4 [55]. Tangeretin (**21**) maintains Nrf2 activation by inhibiting CUL3-mediated ubiquitination, further emphasizing the central role of Nrf2 in antioxidant defense [56]. Additionally, neobavaisoflavone (**8**) protects cells from oxidative stress-induced damage via the CRNDE-mediated Nrf2/HO-1 signaling pathway [57].

Furthermore, a growing body of literature has reported that numerous plant metabolites can improve oxidative stress-induced damage by enhancing the activity of antioxidant enzymes (SOD, HO-1, CAT, and GSH-Px), while simultaneously reducing the levels of malondialdehyde (MDA) and other inflammation-related biomarkers. For instance, some metabolites, such as daidzin (**4**) [58], calycosin (**7**) [59], magnolol (**30**) [60], formononetin (**6**) [61], vitexin (**15**) [62], and isosinensetin (**20**) [39], have been demonstrated to upregulate cellular antioxidant enzyme activities and the expression of various antioxidant enzymes, thereby alleviating oxidative stress-induced damage.

The NF-κB signaling pathway also plays a crucial role in the oxidative stress response. As a major intracellular pro-inflammatory transcription factor, NF-κB mediates inflammatory responses by regulating the expression of a series of pro-inflammatory genes. In the canonical pathway, the NF-κB heterodimer p65/p50 binds to the inhibitory protein IκB, maintaining an inactive state. Upon inflammatory stimuli or oxidative stress, the IκB kinase (IKK) complex activates, phosphorylates and degrades IκB, releasing the NF-κB complex (p65/p50), which is translocated to the nucleus to initiate the expression of inflammatory genes. Studies have shown that rhoifolin (**14**) significantly alleviates ethanol-induced liver damage in mice by inhibiting the TLR4/NF-κB signaling pathway [63]. Additionally, glycitin (**3**) protects lung tissue from LPS-induced inflammation via inhibiting the TLR4-mediated NF-κB and MAPKs signaling pathways [64].

**Figure 5:**
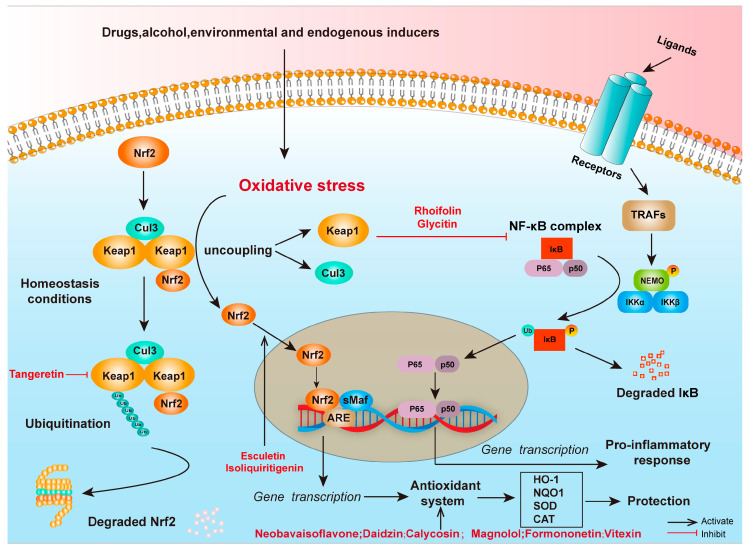
Secondary metabolites contained in tubers and peels of *P. erosus* that exhibit significant antioxidant and anti-inflammatory activities via modulating the Nrf2-Keap1 and NF-κB signaling pathways.

Collectively, the secondary metabolites that were contained in the ethyl acetate fraction of tubers and peels from *P. erosus* exhibited significant antioxidant and anti-inflammatory activities by modulating the Nrf2-Keap1 and NF-κB signaling pathways (Figure 5). These results provide scientific evidence for the application of *P. erosus* extracts in health promotion and drug development. Future studies should explore more natural products with potential applications by integrating the regulatory mechanisms of the Nrf2-Keap1 and NF-κB pathways.

## 5. Conclusions

This study is the first to systematically study the antioxidant activities and phytochemical profiles of petroleum ether, ethyl acetate, n-butanol, and aqueous fractions of the edible (tubers) and non-edible portions (peels) of *P. erosus*. Specifically, the ethyl acetate fraction was identified as the most active fraction in both the peels and tubers, and their chemical constituents were analyzed and identified. Thirty potential metabolites were screened, including twenty-one flavonoid compounds (ten isoflavones, six flavonoid glycosides, three chalcones, two methoxy flavones), six phenolic compounds, two coumarins, and one lignan. Some of these metabolites (flavonoids and phenolic compounds) existed in both the tubers and peels, and have been revealed to exert significant antioxidant effects through the Nrf2-Keap1 and NF-κB signaling pathways. Notably, the peels of *P. erosus*, often considered agricultural waste, showed better antioxidant activity than the tubers in our study. These findings not only provide a reference for the further development and utilization of *P. erosus*, but also suggest a new direction for the utilization of the peels of *P. erosus*, indicating its potential as a natural antioxidant in the food, cosmetic, and pharmaceutical industries.

## Figures and Tables

**Figure 4:**
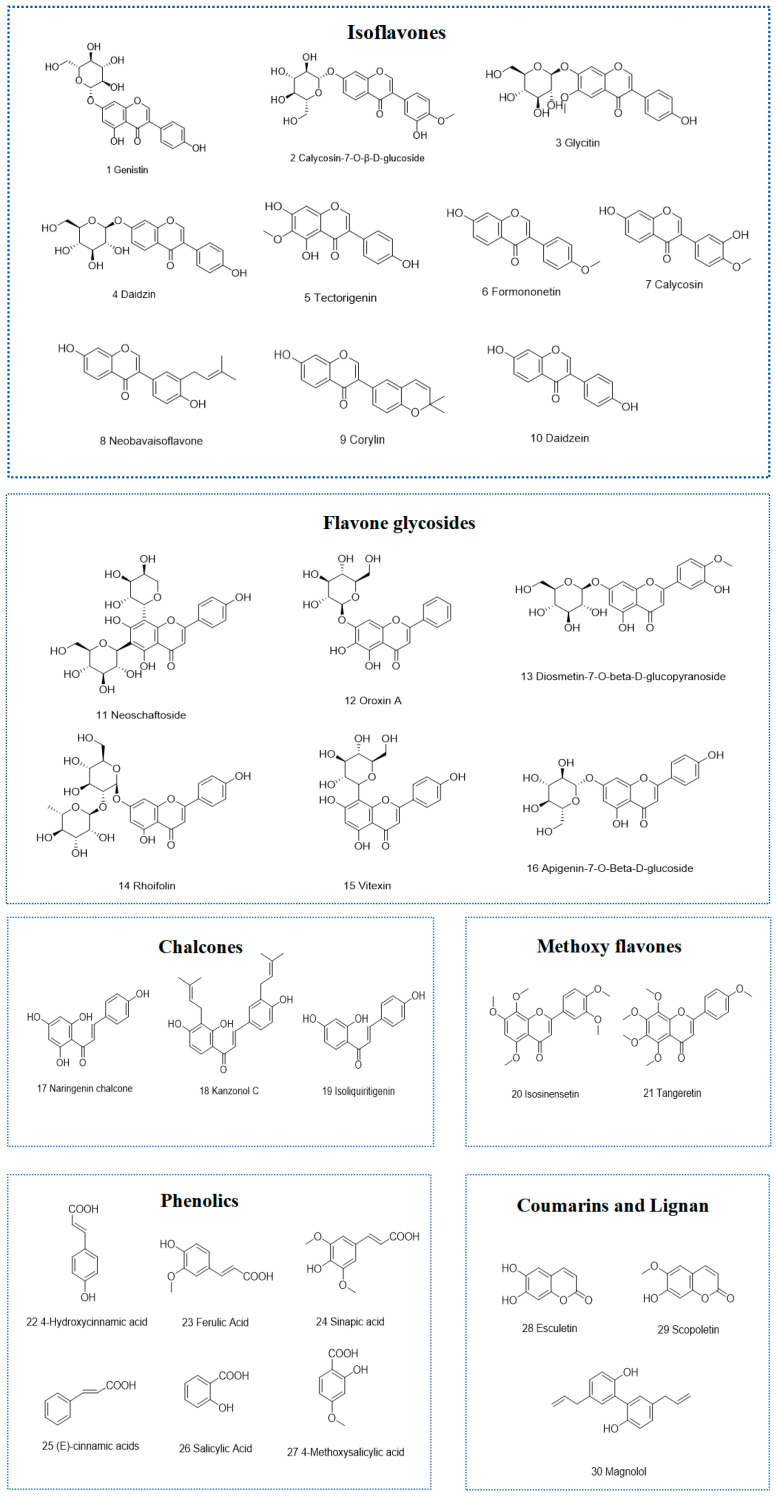
Compounds in the EtOAc fraction of peels and tubers from *P. erosus*.

**Table 1:** The FRAP values of tuber and peel extracts from *P. erosus*.

FRAP (mM Fe (II)/g Extract)	PE	EtOAc	n-BuOH	Aqueous	Standard Curve
Peel	7.023 ± 0.5591	12.56 ± 1.720	2.519 ± 0.4109	0.6443 ± 0.09023	Y = 0.4196x + 0.0023, R^2^ = 0.9997
Tuber	3.242 ± 0.8131	4.113 ± 1.158	1.200 ± 0.2784	0.3636 ± 0.1828	Y = 0.4196x + 0.0023, R^2^ = 0.9997

Data are expressed as mean ± SD (n = 3).

**Table 2:** Identification of chemical constituents of the EtOAc fraction of peels and tubers from *P. erosus* by UPLC-Orbitrap-MS/MS.

No.	Compound Name	tR (min)	Formula	Mode	Mass Error (ppm)	*m*/*z*
**Isoflavones**						
**1**	Genistin	5.000733333	C_21_H_20_O_10_	pos	−1.436209521	433.1123027
**2**	Calycosin-7-O-β-D-glucoside	5.482933333	C_22_H_22_O_10_	pos	−1.394990775	447.127951
**3**	Glycitin	5.653616667	C_22_H_22_O_10_	pos	−1.516097102	447.127897
**4**	Daidzin	5.684666667	C_21_H_20_O_9_	neg	−0.120060599	415.1034058
**5**	Tectorigenin	6.809166667	C_16_H_12_O_6_	neg	−0.613314336	299.0559276
**6**	Formononetin	8.593933333	C_16_H_12_O_4_	neg	−0.38949262	267.066178
**7**	Calycosin	7.305166667	C_16_H_12_O_5_	pos	−2.282324897	285.0751016
**8**	Neobavaisoflavone	9.39485	C_20_H_18_O_4_	pos	−2.253131166	323.1270597
**9**	Corylin	10.12973333	C_20_H_16_O_4_	neg	−0.747793006	319.0973432
**10**	Daidzein	15.10716667	C_15_H_10_O_4_	neg	−0.162841595	253.050591
**Flavone glycosides**						
**11**	Neoschaftoside	5.23475	C_26_H_28_O_14_	neg	−0.008722792	563.1406242
**12**	Oroxin A	5.246433333	C_21_H_20_O_10_	neg	−0.900136026	431.0979815
**13**	Diosmetin-7-O-Beta-D-glucopyranoside	5.26775	C_22_H_22_O_11_	neg	0.220291863	461.1090369
**14**	Rhoifolin	5.73185	C_27_H_30_O_14_	neg	0.192143435	577.1563903
**15**	Vitexin	5.9234	C_21_H_20_O_10_	pos	−1.766953107	433.1121598
**16**	Apigenin-7-O-Beta-D-glucoside	6.586366667	C_21_H_20_O_10_	neg	−0.461695408	431.0981709
**Chalcones**						
**17**	Naringenin chalcone	7.83725	C_15_H_12_O_5_	pos	−2.643945598	273.0750306
**18**	Kanzonol C	8.081333333	C_25_H_28_O_4_	neg	−6.316384301	391.1890057
**19**	Isoliquiritigenin	8.369116667	C_15_H_12_O_4_	neg	0.041119868	255.066293
**Methoxy flavones**						
**20**	Isosinensetin	8.305783333	C_20_H_20_O_7_	pos	−2.241569305	373.1273453
**21**	Tangeretin	9.87585	C_20_H_20_O_7_	pos	−2.043774215	373.1274189
**Phenolics**						
**22**	4-Hydroxycinnamic acid	5.785916667	C_9_H_8_O_3_	neg	−0.108469542	163.0400499
**23**	Ferulic acid	6.00555	C_10_H_10_O_4_	pos	−1.140024779	195.064964
**24**	Sinapinic acid	6.014833333	C_11_H_12_O_5_	neg	−0.173561152	223.0611582
**25**	(E)-Cinnamic acid	6.185683333	C_9_H_8_O_2_	neg*	−0.734750116	193.0505236
**26**	Salicylic acid	6.251733333	C_7_H_6_O_3_	neg	−0.687050513	137.0243228
**27**	4-Methoxysalicylic acid	7.014166667	C_8_H_8_O_4_	neg	−0.327807242	167.0349272
**Coumarins**						
**28**	Esculetin	5.108033333	C_9_H_6_O_4_	neg	−0.58725632	177.0192277
**29**	Scopoletin	6.094333333	C_10_H_8_O_4_	neg	0.026476372	191.0349874
**Lignan**						
**30**	Magnolol	10.99731667	C_18_H_18_O_2_	neg	−0.277636992	265.1233295

neg*: neg (M + FA − H), FA = HCOOH.

**Table 3:** Secondary metabolites contained in tubers and peels of *P. erosus* that exhibit significant pharmacological activities.

Compound Name	Activity	References
Genistin (**1**)	Cardioprotective; Anti-diabetes	[23]
Calycosin-7-O-β-D-glucoside (**2**)	Anti-diabetes	[24]
Glycitin (**3**)	Anti-obese; Anti-diabetes	[25]
Daidzin (**4**)	Anti-epileptic	[23]
Tectorigenin (**5**)	Neuroprotective effect; Anti-diabetic cardiomyopathy	[23]
Formononetin (**6**)	Anti-gastric ulcers; Anti-cancer	[23]
Calycosin (**7**)	Anti-cancer; Neuroprotective effect	[26,27]
Neobavaisoflavone (**8**)	Neuroprotective effect	[28]
Corylin (**9**)	Anti-cancer	[29]
Daidzein (**10**)	Anti-obesity; Neuroprotective effect	[5,23]
Oroxin A (**12**)	Anti-cancer; Anti-diabetes	[30,31]
Rhoifolin (**14**)	Anti-cancer	[32]
Vitexin (**15**)	Anti-cancer; Anti-depressant	[33,34]
Naringenin chalcone (**17**)	Anti-cancer; Anti-allergic	[35,36]
Isoliquiritigenin (**19**)	Anti-cancer; Anti-diabetes	[37,38]
Isosinensetin (**20**)	Anti-Osteoporosis	[39]
Tangeretin (**21**)	Anti-cancer; Anti-microbia	[40,41]
4-Hydroxycinnamic acid (**22**)	Neuroprotective effect	[42]
Ferulic acid (**23**)	Antimicrobial	[43,44]
Sinapinic acid (**24**)	Anti-thrombosis; Anti-cancer; Cardioprotective; Anti-convulsant	[45,46,47,48]
Salicylic acid (**26**)	Anti-fungal	[49]
Esculetin (**28**)	Anti-tumor; Anti-diabetes	[50]
Scopoletin (**29**)	Anti-bacterial; Anti-fungal	[51]
Magnolol (**30**)	Anti-gastritic; Anti-fungal	[52,53]

## Data Availability

Data will be made available on request from the corresponding author.

## Supplementary Materials

The following supporting information can be downloaded at: https://www.mdpi.com/article/10.3390/antiox14040416/s1, Figure S1: Base peak chromatogram of the EtOAc fraction of peels and tubers from *P. erosus*; Figures S2–S31: MS/MS2 spectrum of compounds **1–30**; Figure S32: PCA scores plot showing the relationship among TFC, TPC, and antioxidant activity (DPPH, ABTS, FRAP). Table S1: 30 compounds screened from the highly active EtOAc fraction of *P. erosus* peel and tuber according to the scoring.
