# Supplementary material for: Phytochemical Profile and Antioxidant Activity of the Tuber and Peel of Pachyrhizus erosus
Source: Antioxidants (Basel). 2025 Mar 31;14(4):416. doi: 10.3390/antiox14040416 (PMC12023986; doi:10.3390/antiox14040416)
Supplement: Supplementary file 1 [file antioxidants-14-00416-s001.zip › Supplementary material-Fig S1-S32.pdf]

Supplementary material

The List of Contents

No.	Contents	Page
1	**Figure S1.** Base peak chromatogram of strongly active fractions in the EtOAc phase from 95% ethanol extracts of *Pachyrhizus erosus*.	S2
2	**Figure S2-S11**. MS/MS2 spectrum of isoflavones	S4
3	**Figure S12-S17**. MS/MS2 spectrum of flavone glycosides	S9
4	**Figure S18-S22**. MS/MS2 spectrum of methoxyflavones and chalcones	S12
5	**Figure S23-S28**. MS/MS2 spectrum of phenolic compounds	S14
6	**Figure S29-S31**. MS/MS2 spectrum of coumarins and lignan	S17
7	**Figure S32**. PCA scores plot showing the relationship among TFC, TPC, and antioxidant activity (DPPH, ABTS, FRAP)	S19

(a)

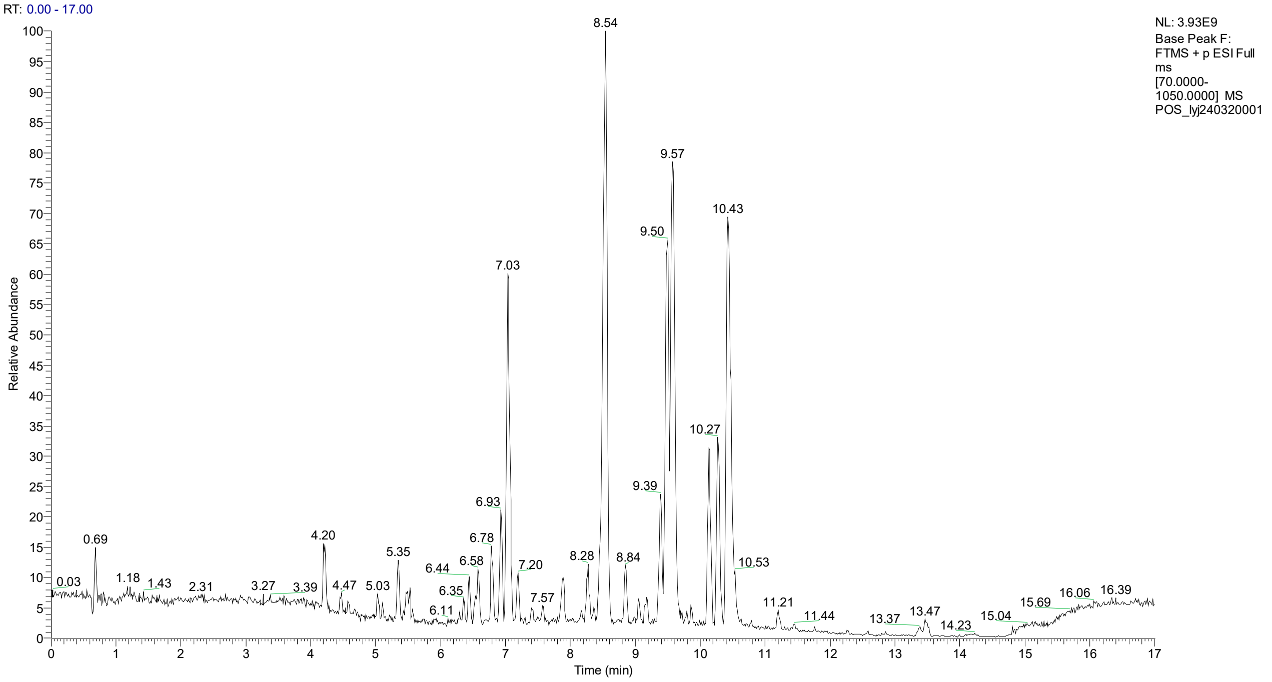
RT: 0.00 - 17.00
NL: 3.93E9
Base Peak F:
FTMS + p ESI Full
ms
[70.0000-
1050.0000] MS
POS_lyj240320001
Relative Abundance
Time (min)

(b)

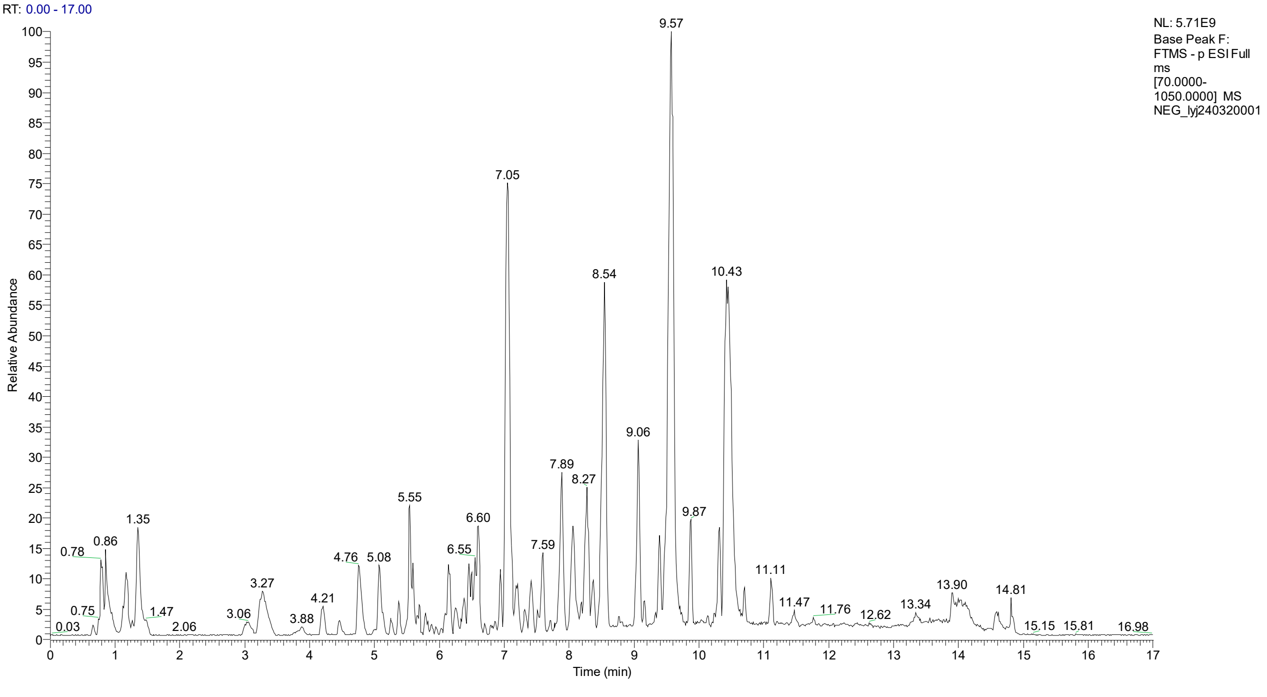
RT: 0.00 - 17.00
NL: 5.71E9
Base Peak F:
FTMS - p ESI Full
ms
[70.0000-
1050.0000] MS
NEG_lyj240320001
Relative Abundance
Time (min)

(c)

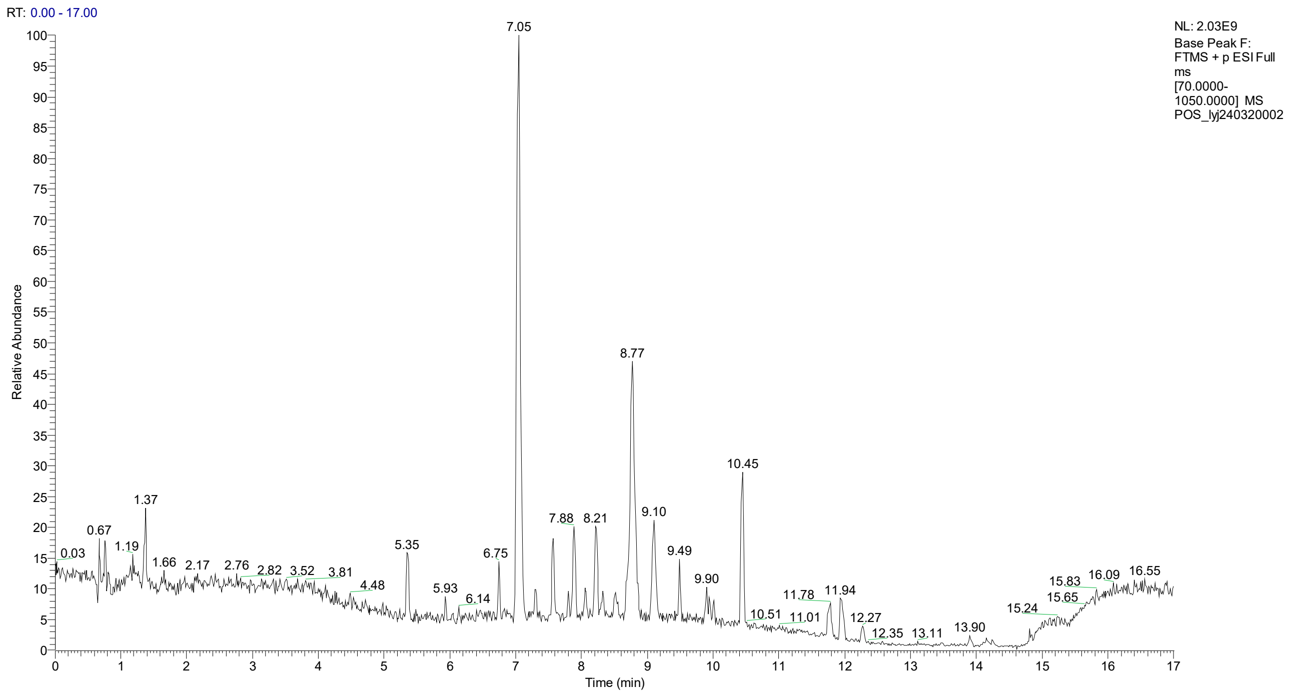

(d)

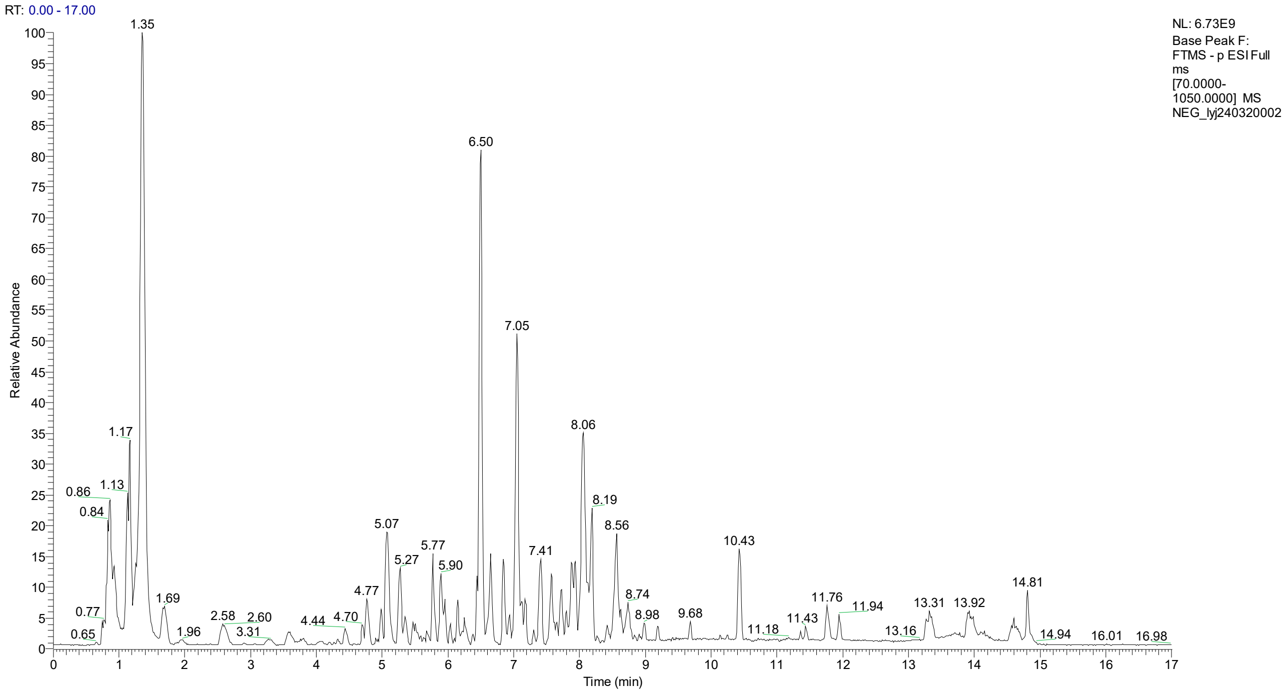

Figure S1. Base peak chromatogram of strongly active fractions in the EtOAc phase from 95% ethanol extracts of *Pachyrhizus erosus*: a. Positive ion mode of tuber, b. Negative ion mode of tuber, c. Positive ion mode of peel, d. Negative ion mode of peel.

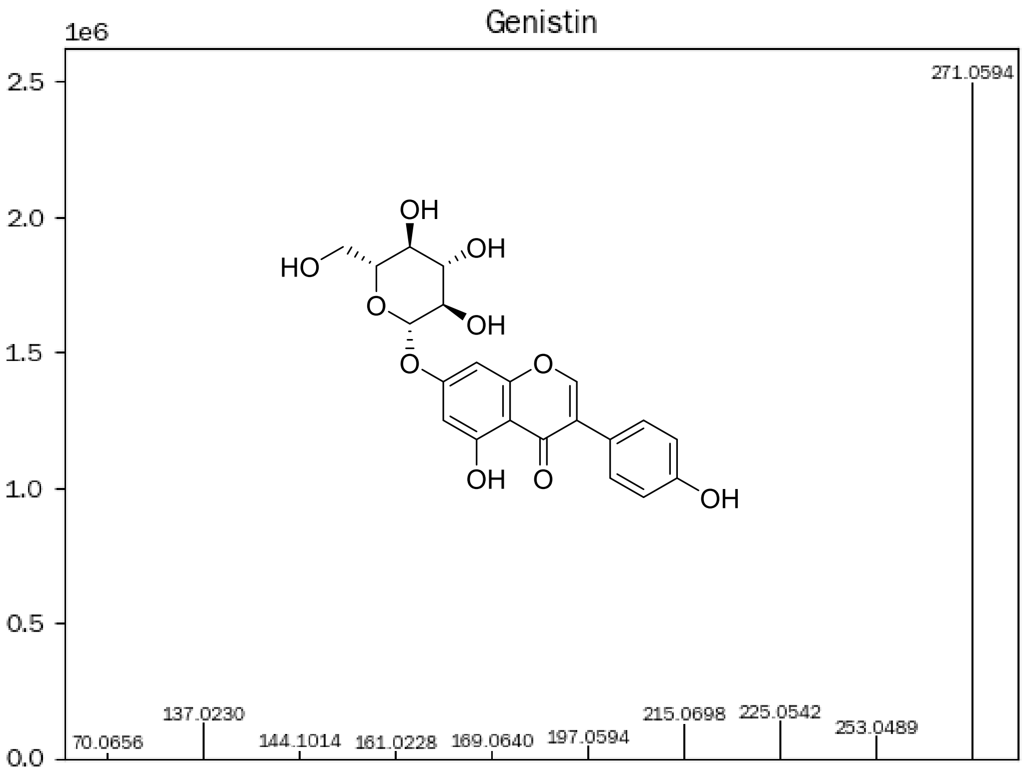

Figure S2. MS/MS2 spectrum of genistin

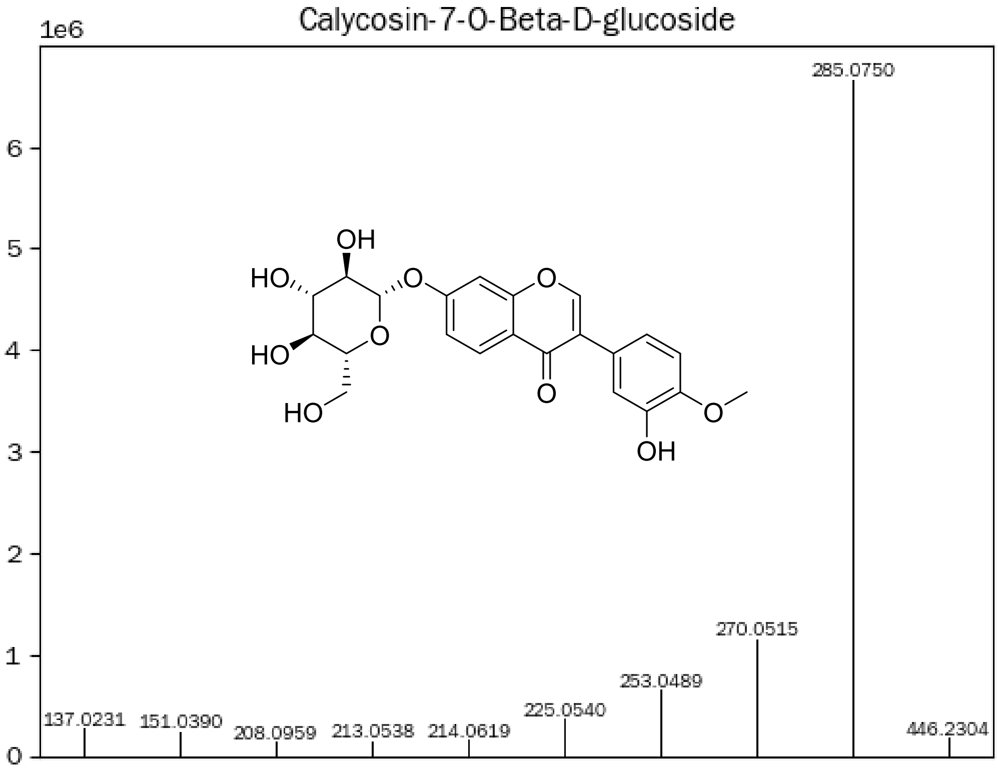

Figure S3. MS/MS2 spectrum of calycosin-7-O-Beta-D-glucoside

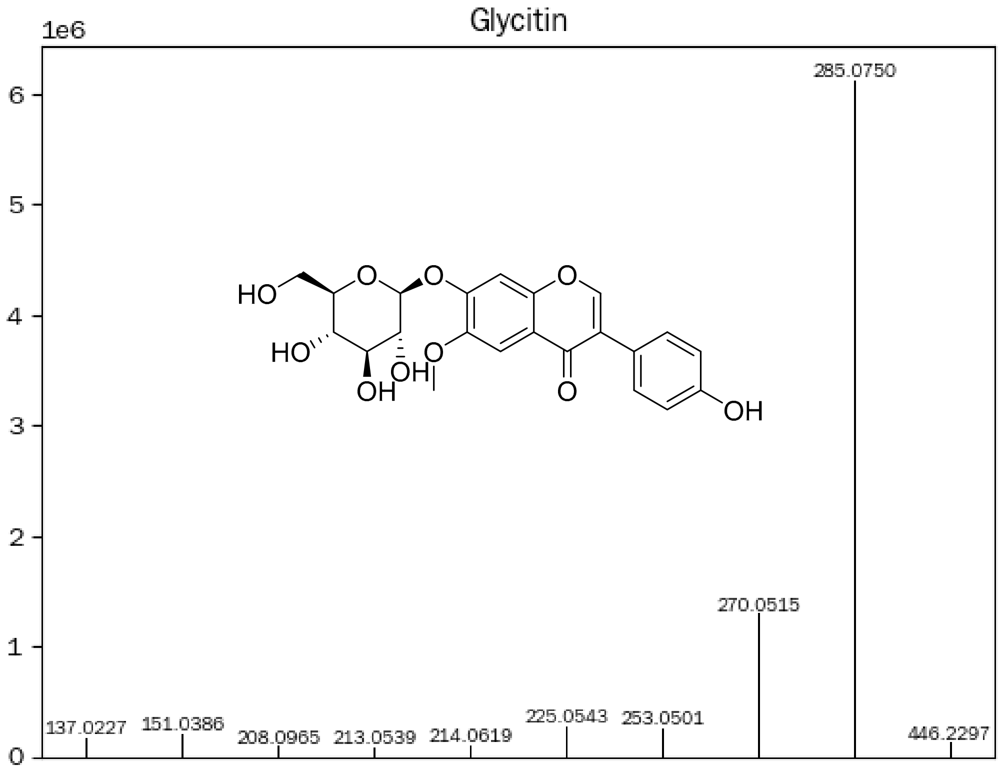

Figure S4. MS/MS2 spectrum of glycitin

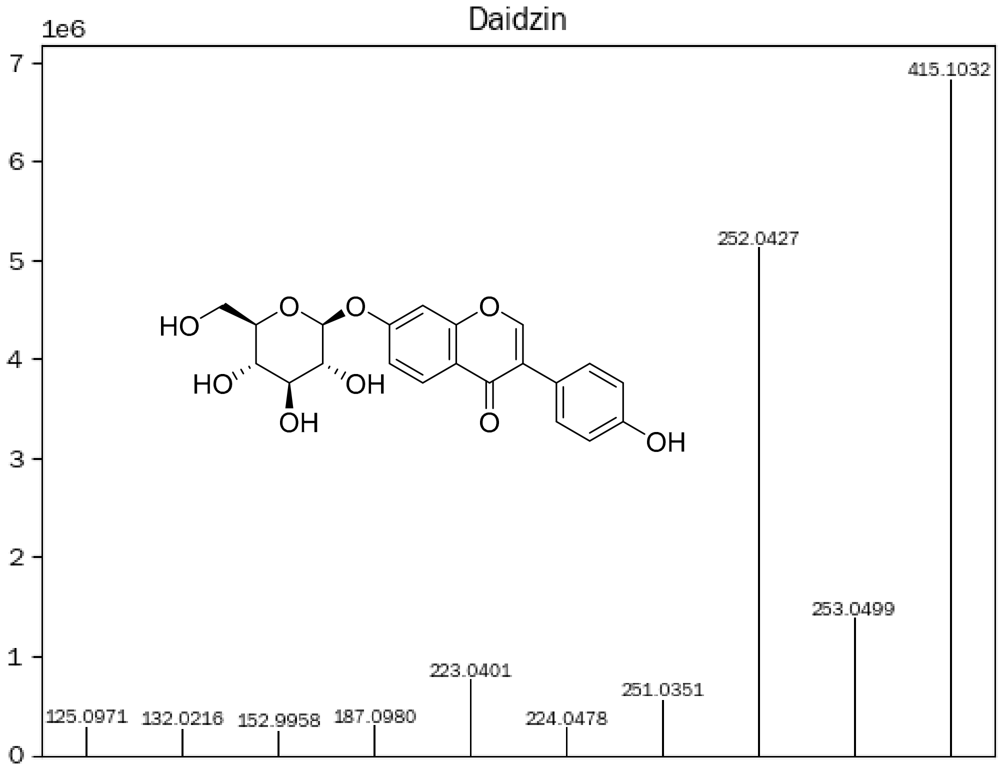

Figure S5. MS/MS2 spectrum of daidzin

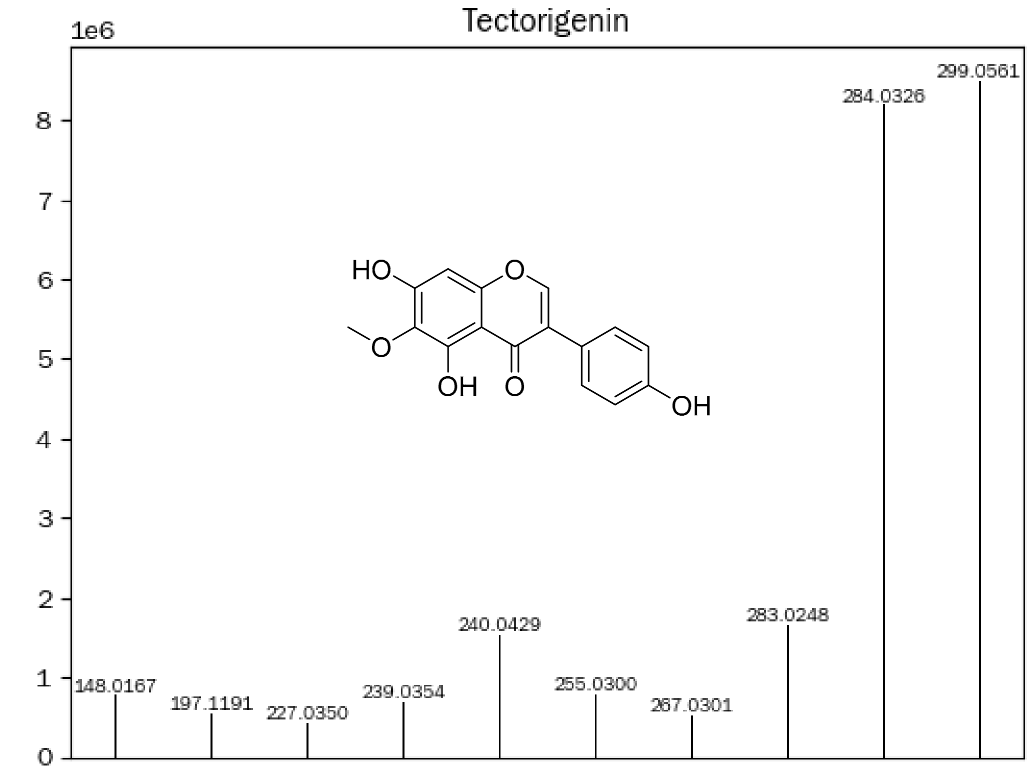

Figure S6. MS/MS2 spectrum of tectorigenin

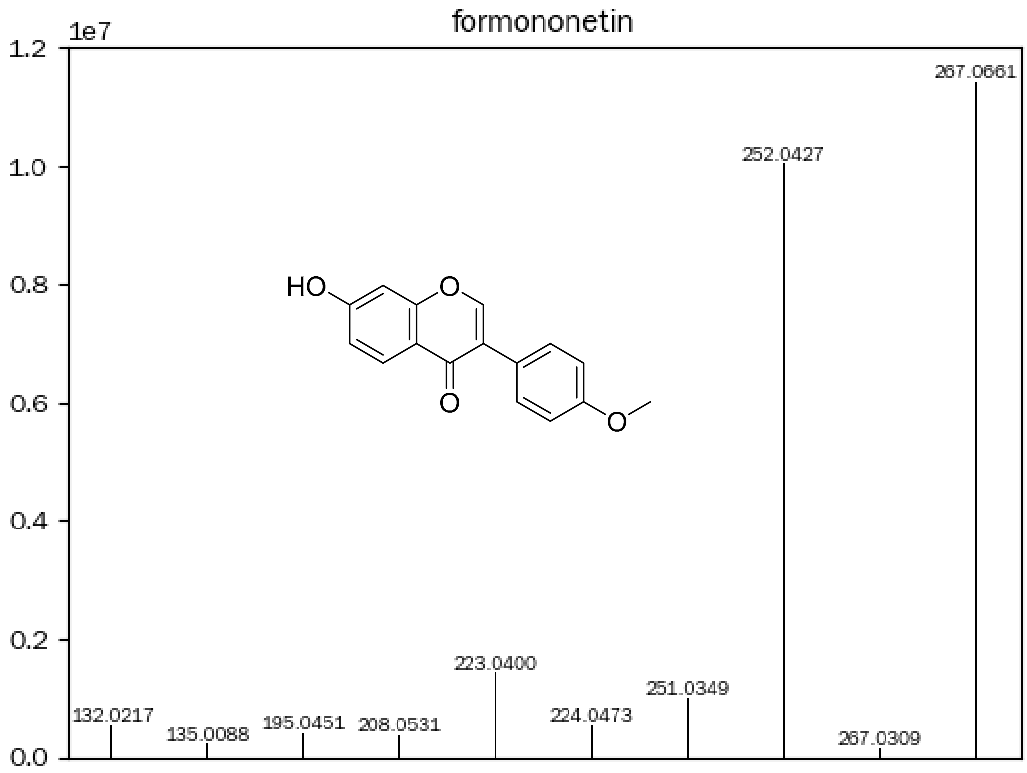

Figure S7. MS/MS2 spectrum of formononetin

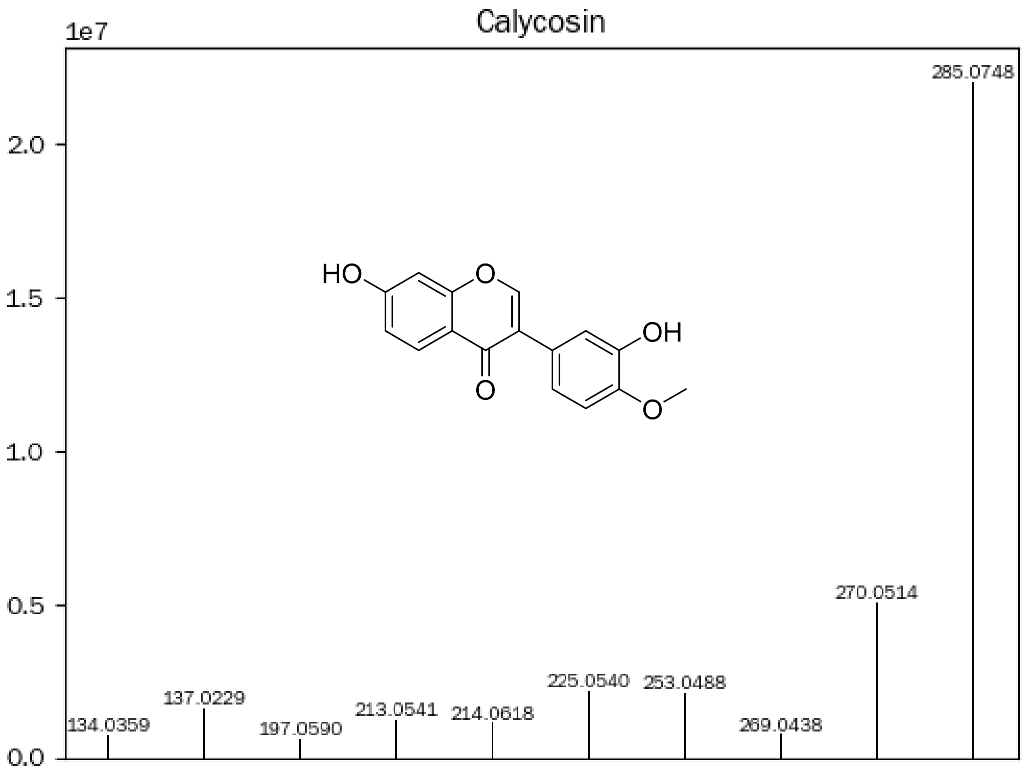

Figure S8. MS/MS2 spectrum of calycosin

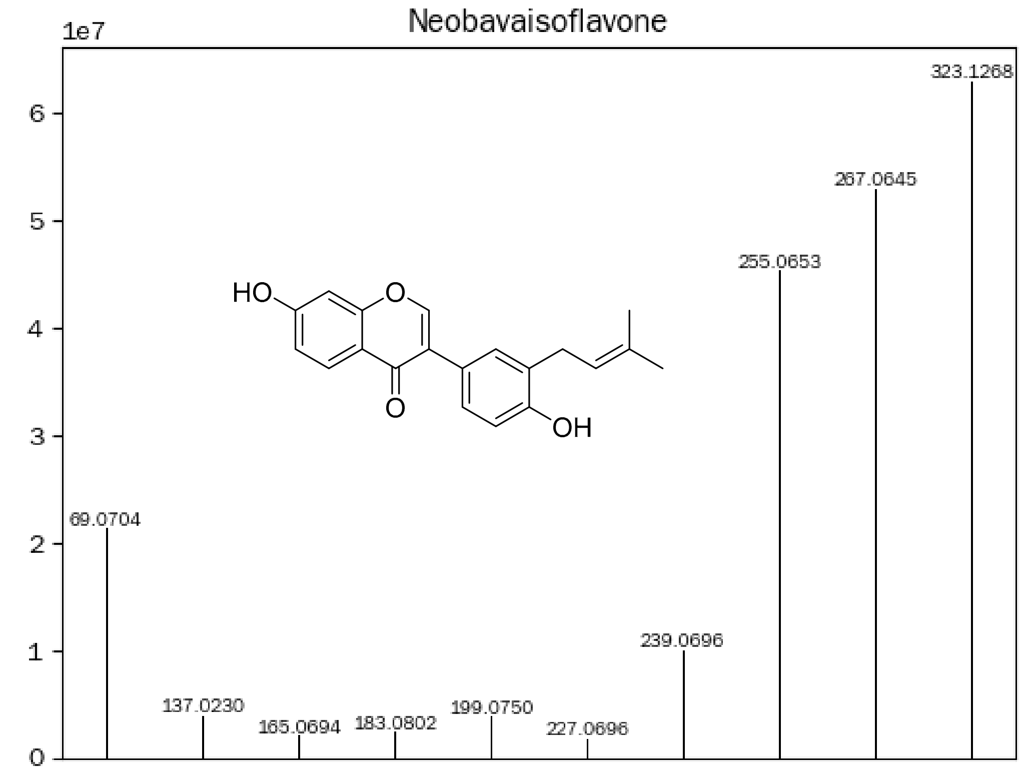

Figure S9. MS/MS2 spectrum of neobavaisoflavone

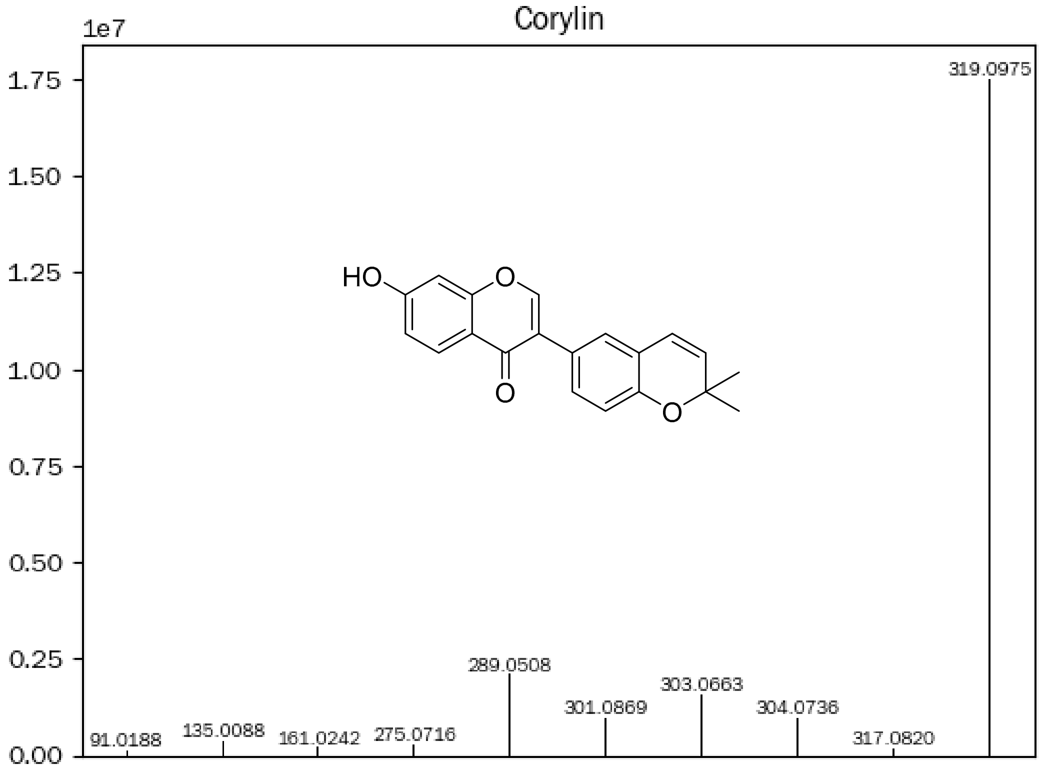

Figure S10. MS/MS2 spectrum of corylin

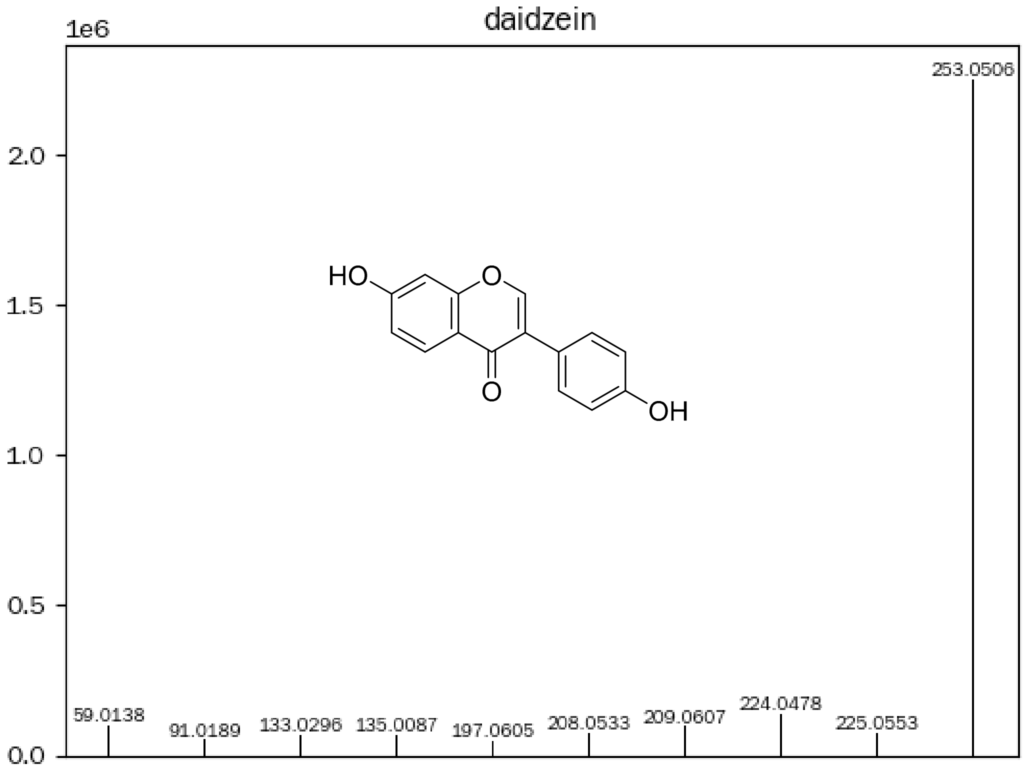

Figure S11. MS/MS2 spectrum of daidzein

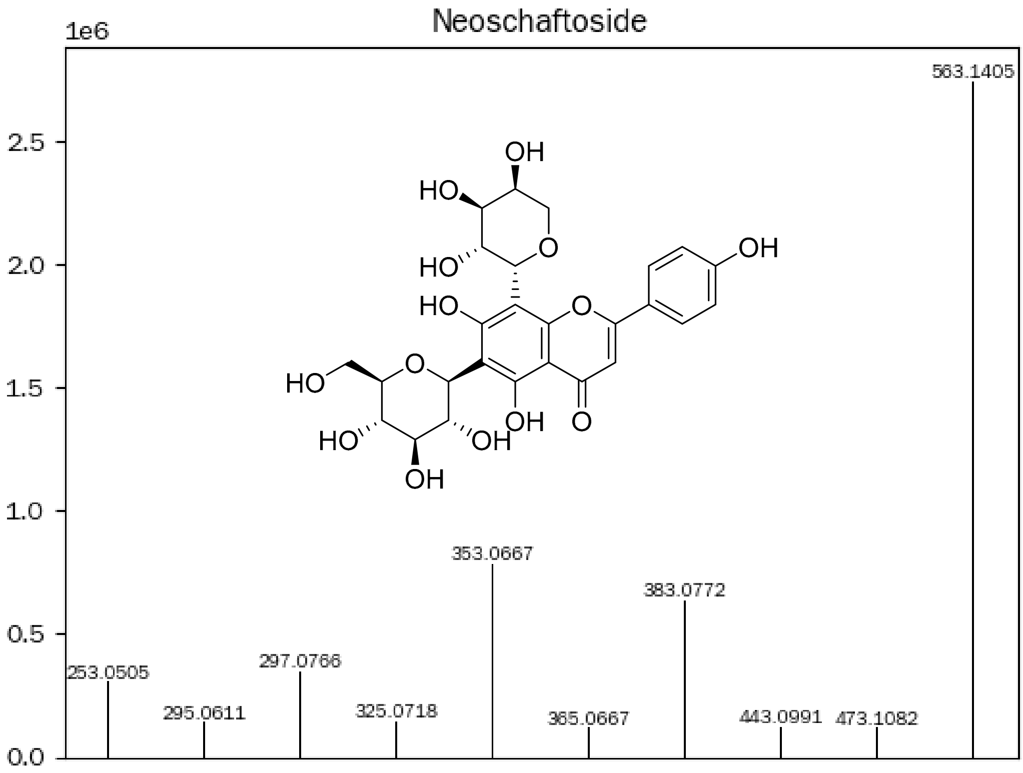

Figure S12. MS/MS2 spectrum of neoschaftoside

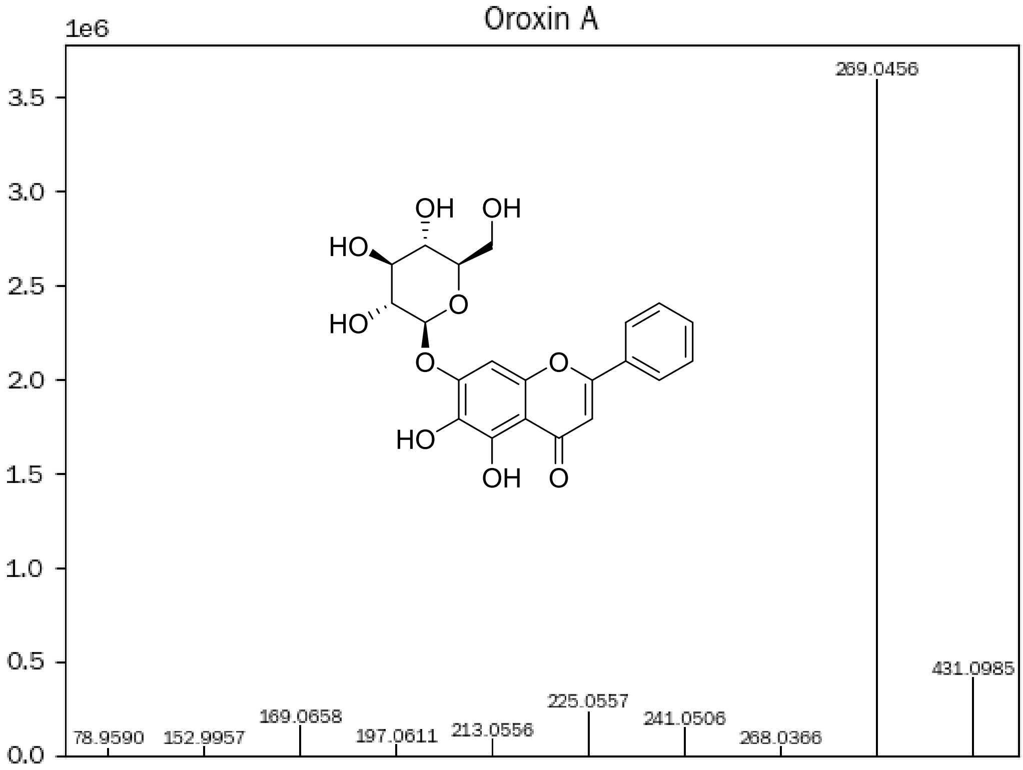

Figure S13. MS/MS2 spectrum of oroxin A

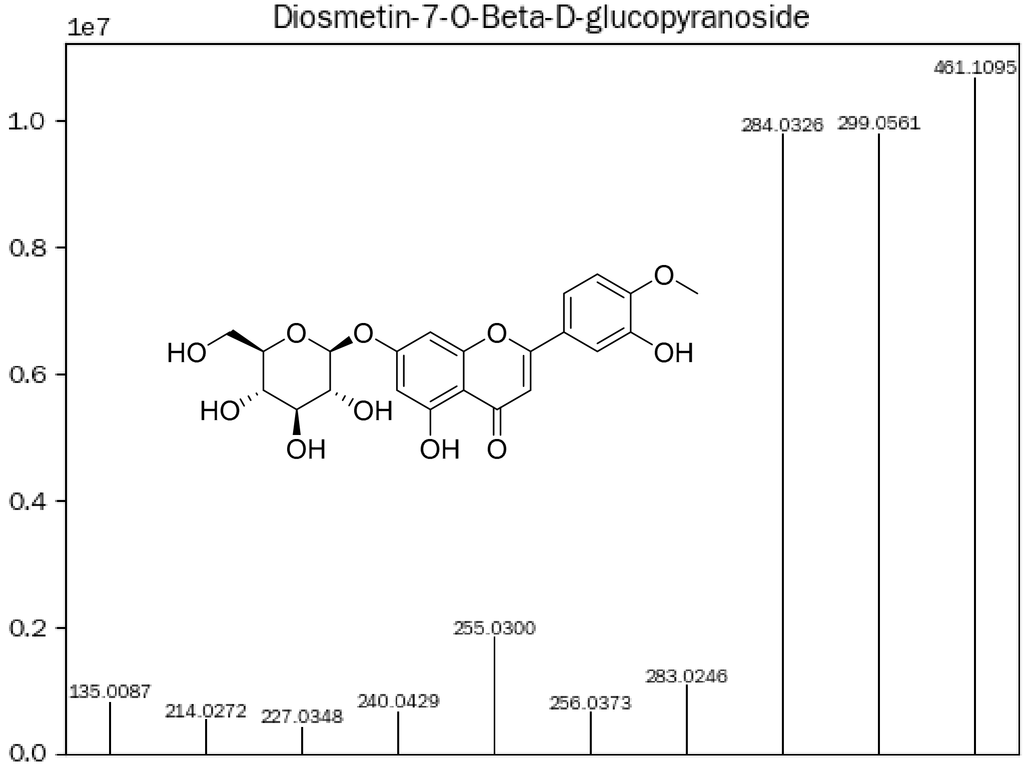

Figure S14. MS/MS2 spectrum of diosmetin-7-O-Beta-D-glucopyranoside

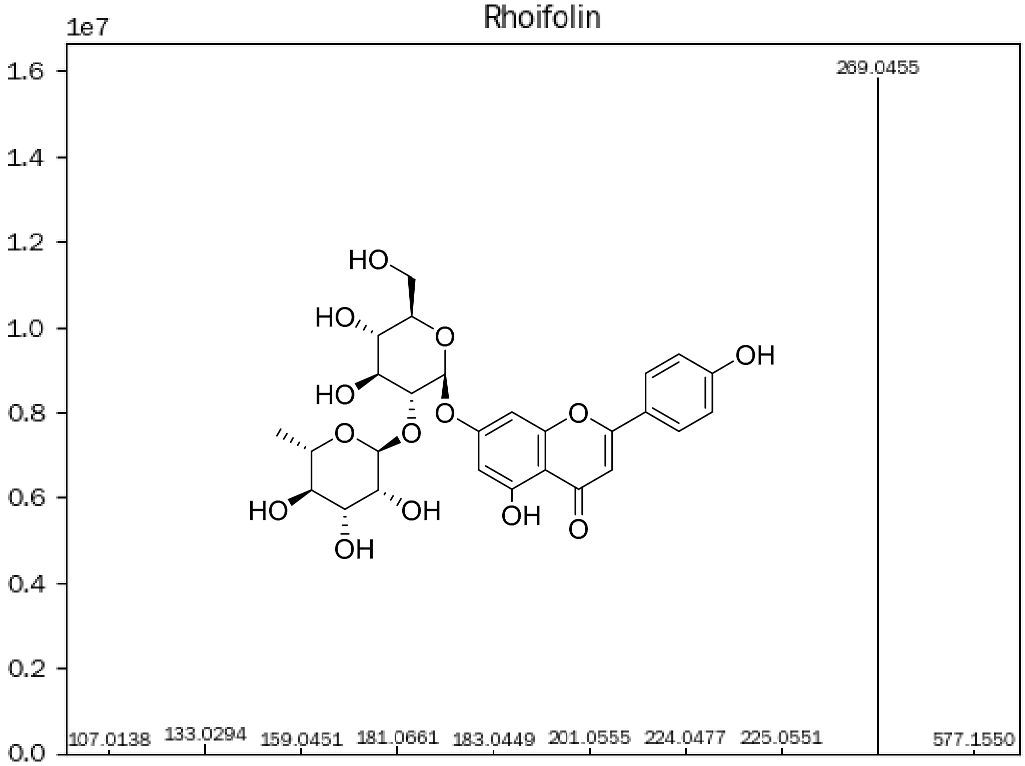

Figure S15. MS/MS2 spectrum of rhoifolin

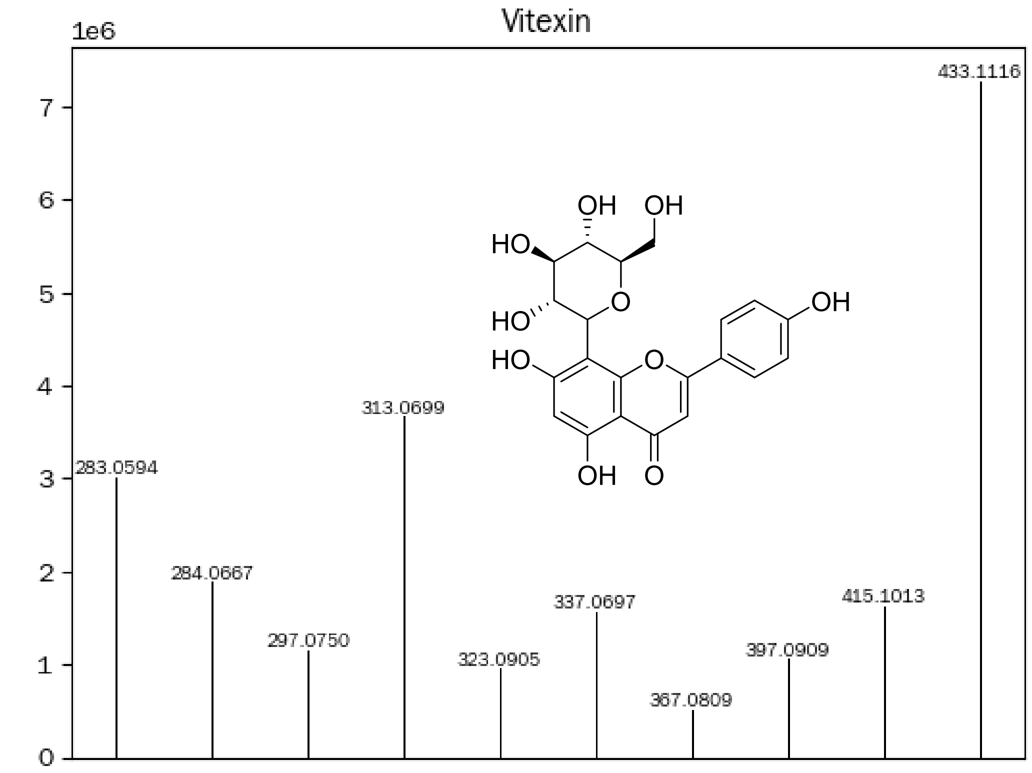

Figure S16. MS/MS2 spectrum of vitexin

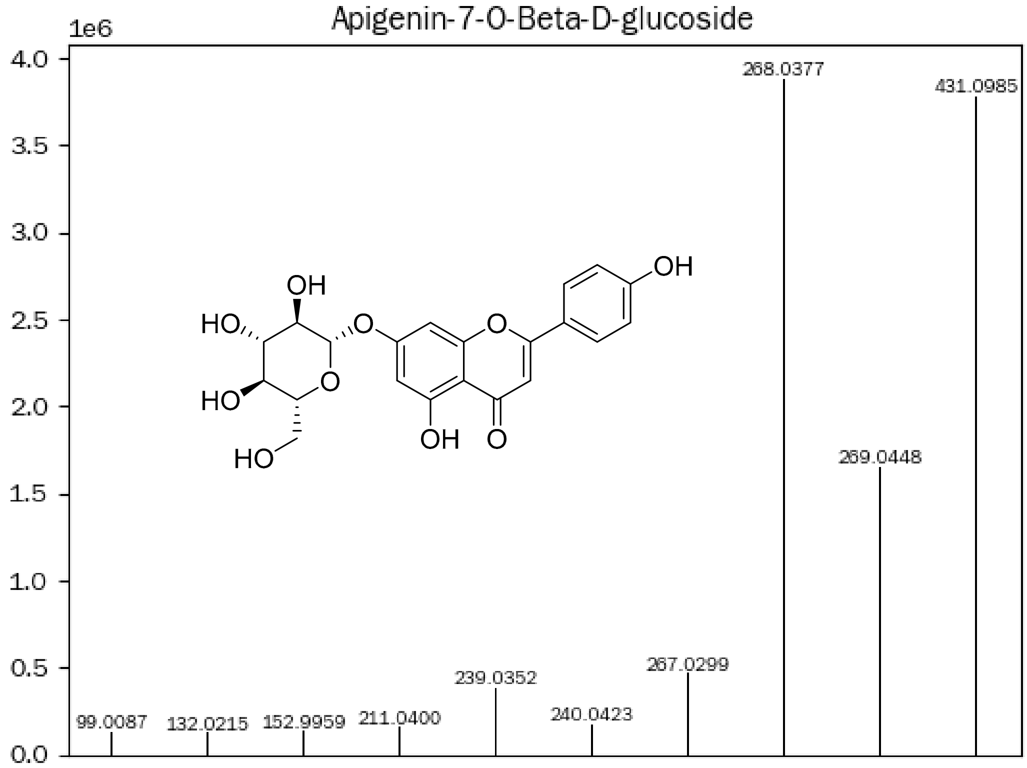

Figure S17. MS/MS2 spectrum of apigenin-7-O-Beta-D-glucoside

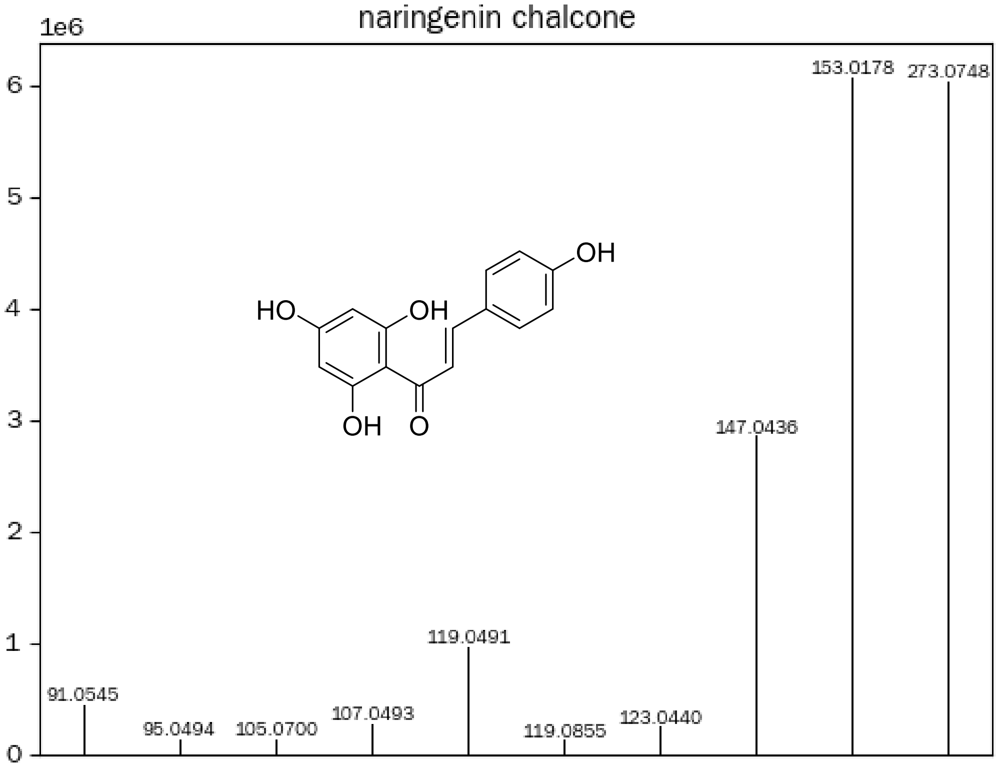

Figure S18. MS/MS2 spectrum of naringenin chalcone

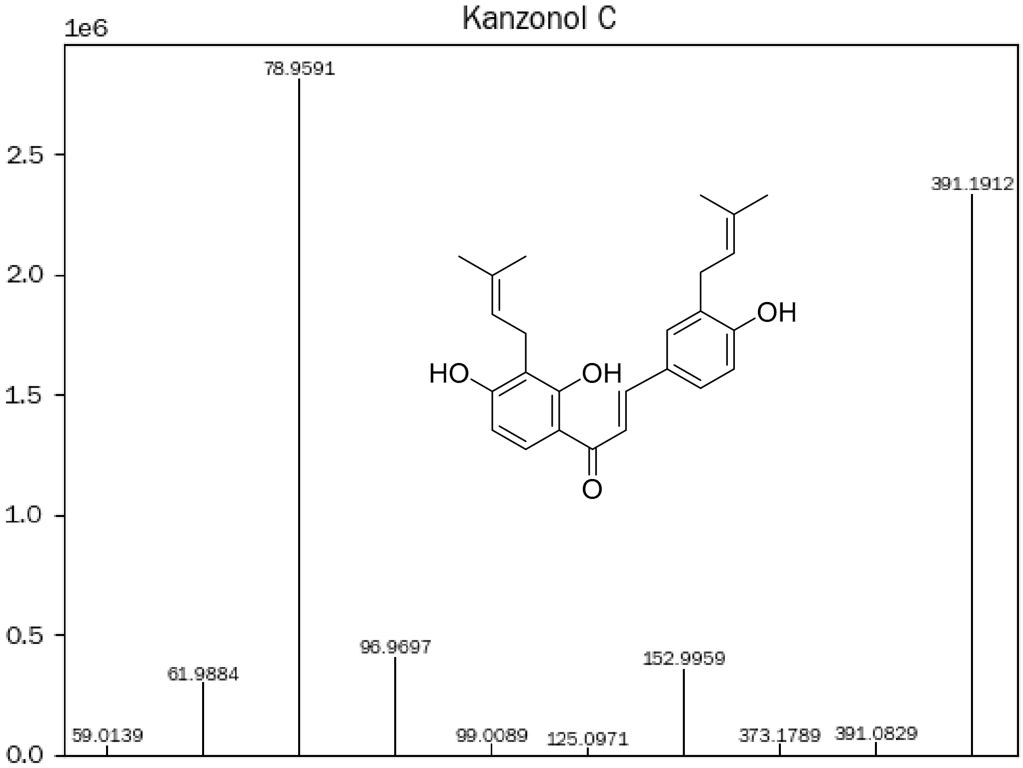

Figure S19. MS/MS2 spectrum of kanzonol C

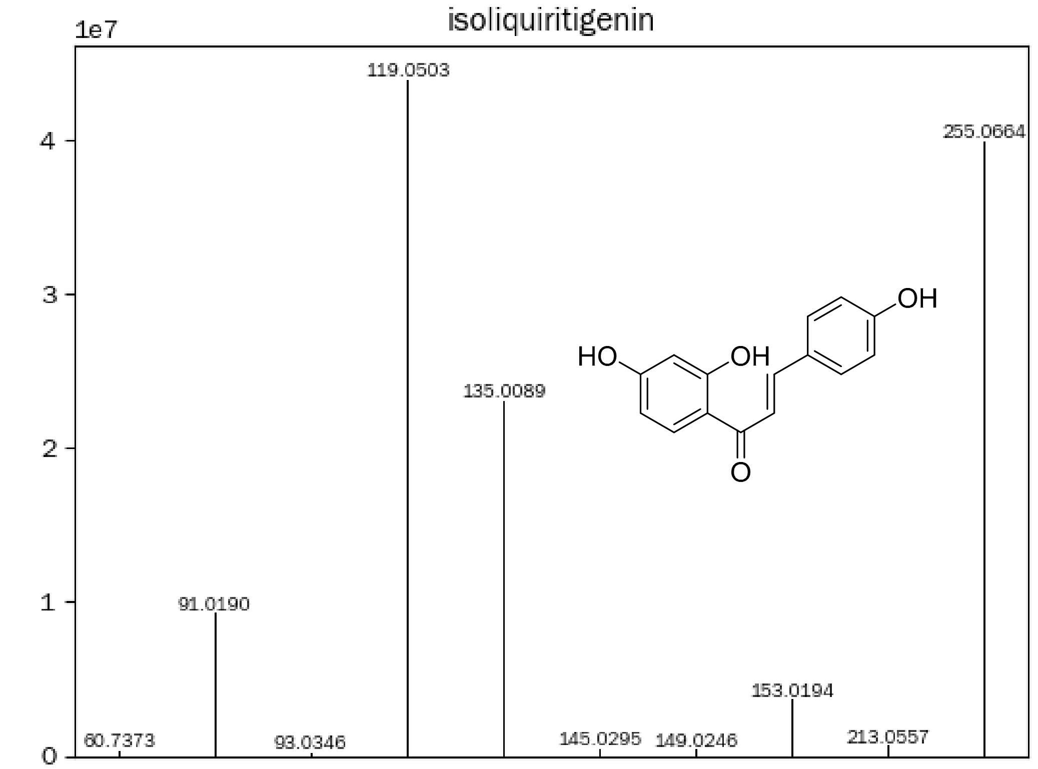

Figure S20. MS/MS2 spectrum of isoliquiritigenin

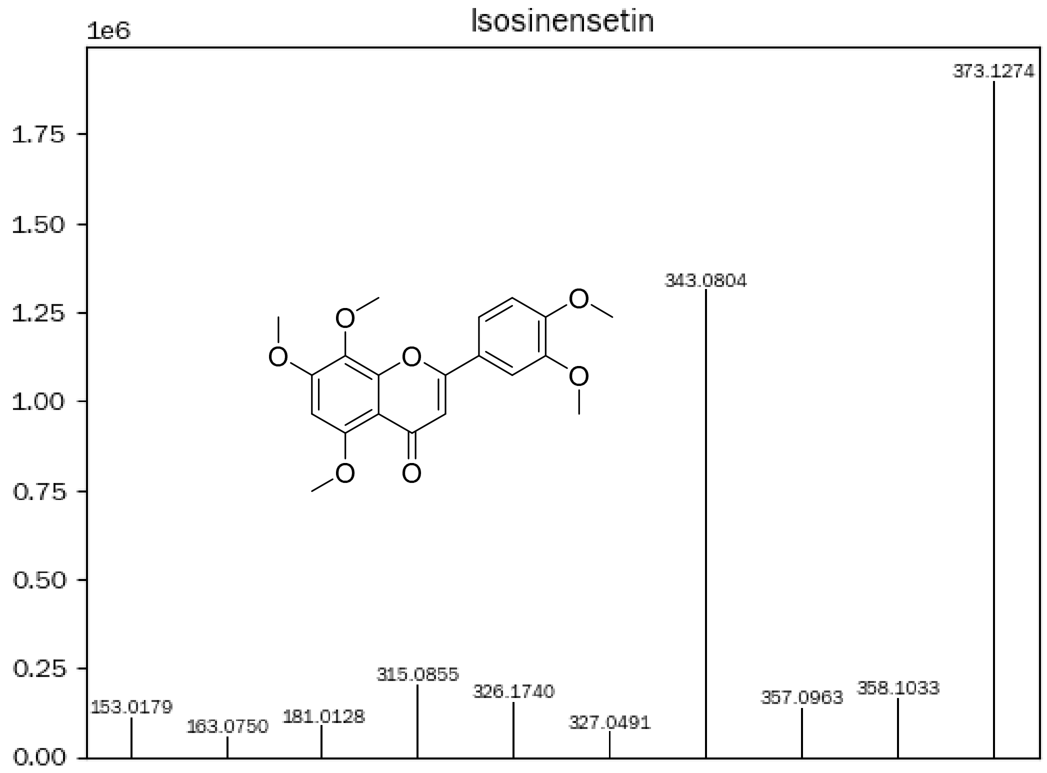

Figure S21. MS/MS2 spectrum of isosinensetin

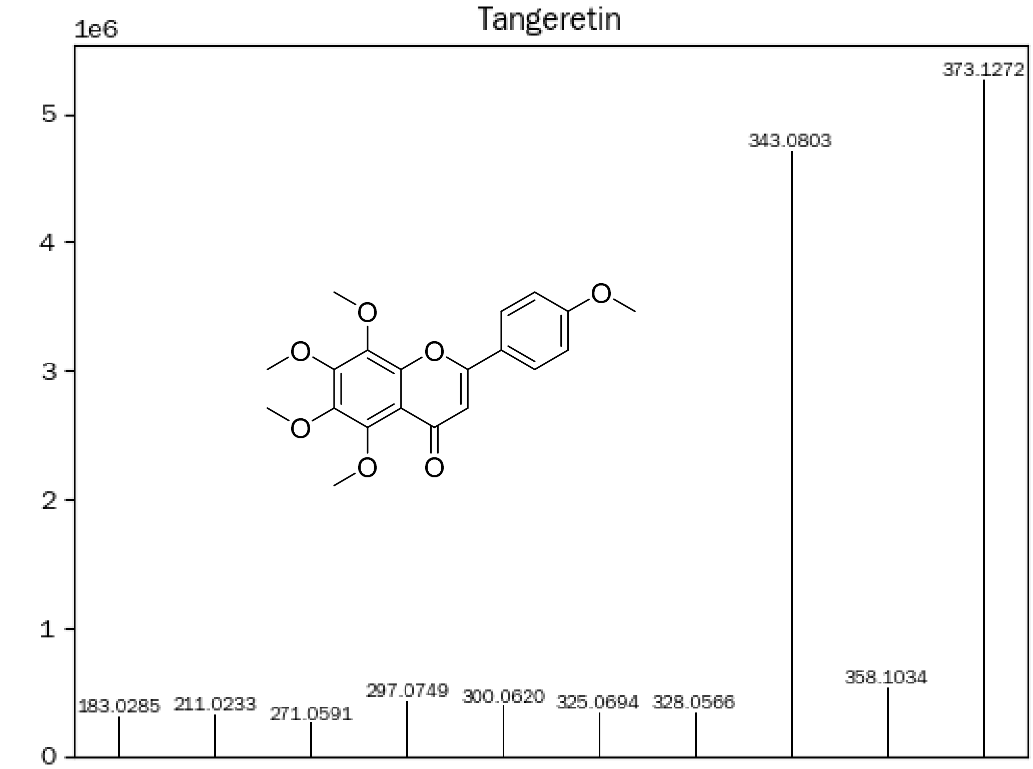

Figure S22. MS/MS2 spectrum of tangeretin

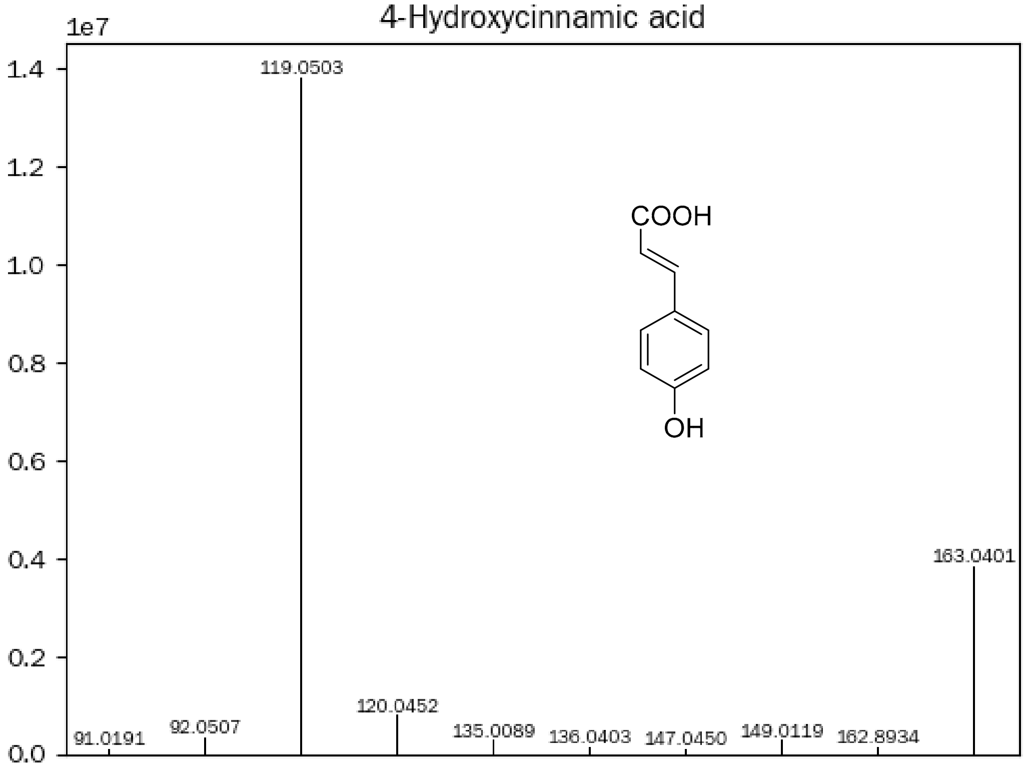

Figure S23. MS/MS2 spectrum of 4-Hydroxycinnamic acid

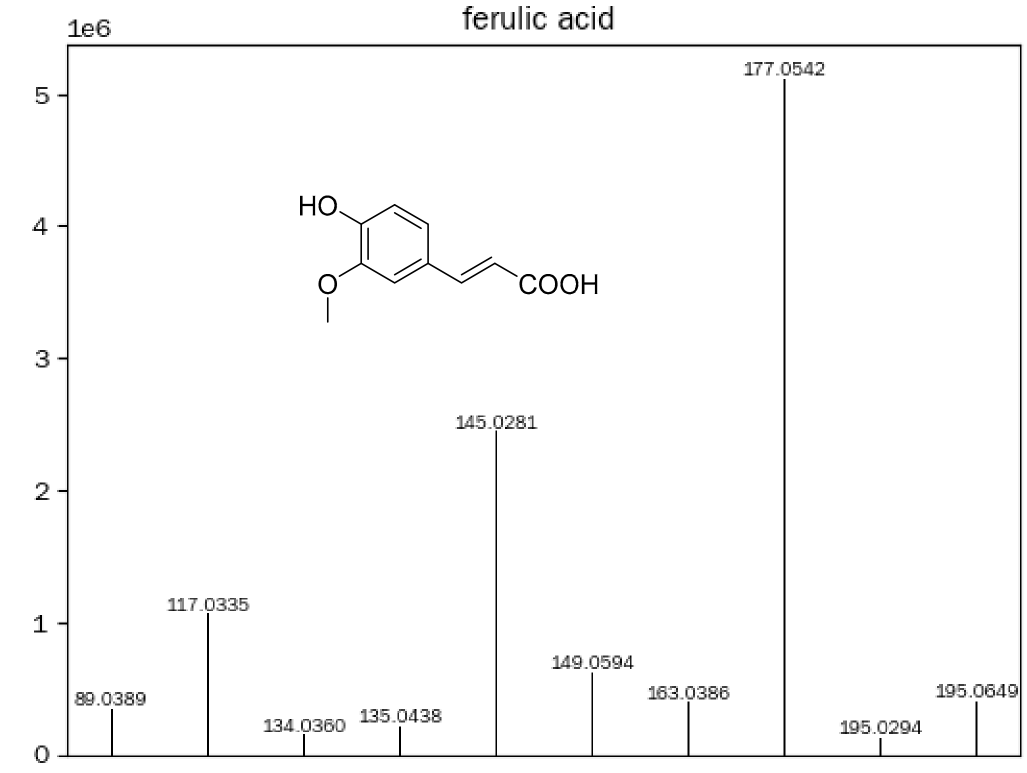

Figure S24. MS/MS2 spectrum of ferulic acid

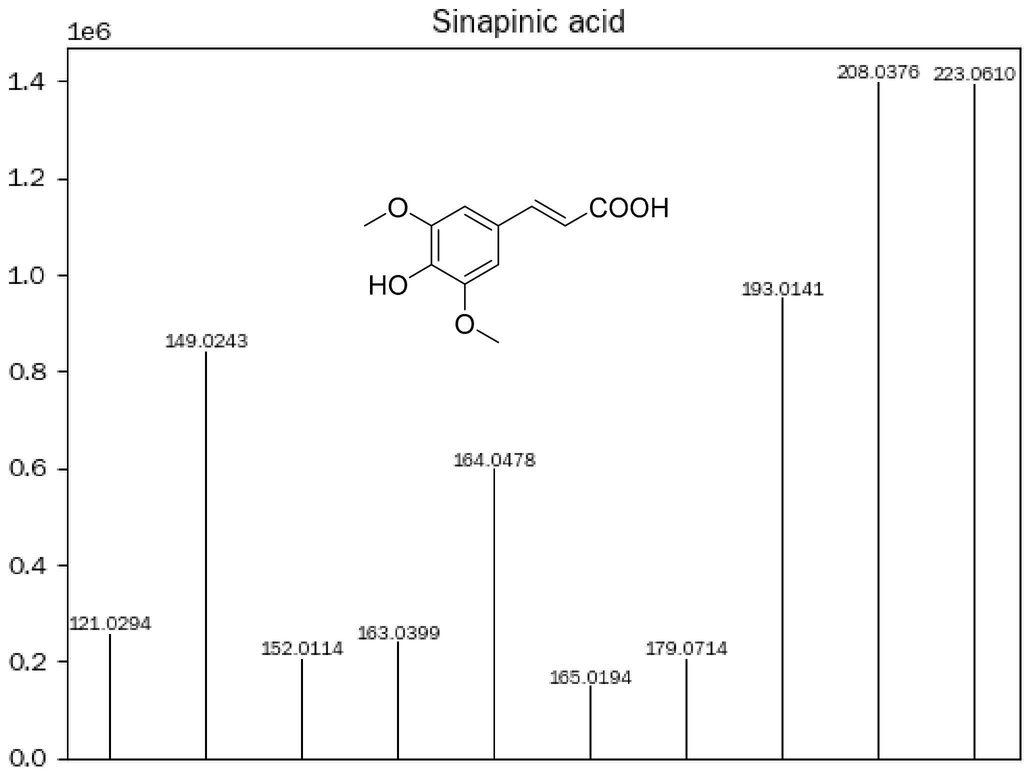

Figure S25. MS/MS2 spectrum of sinapinic acid

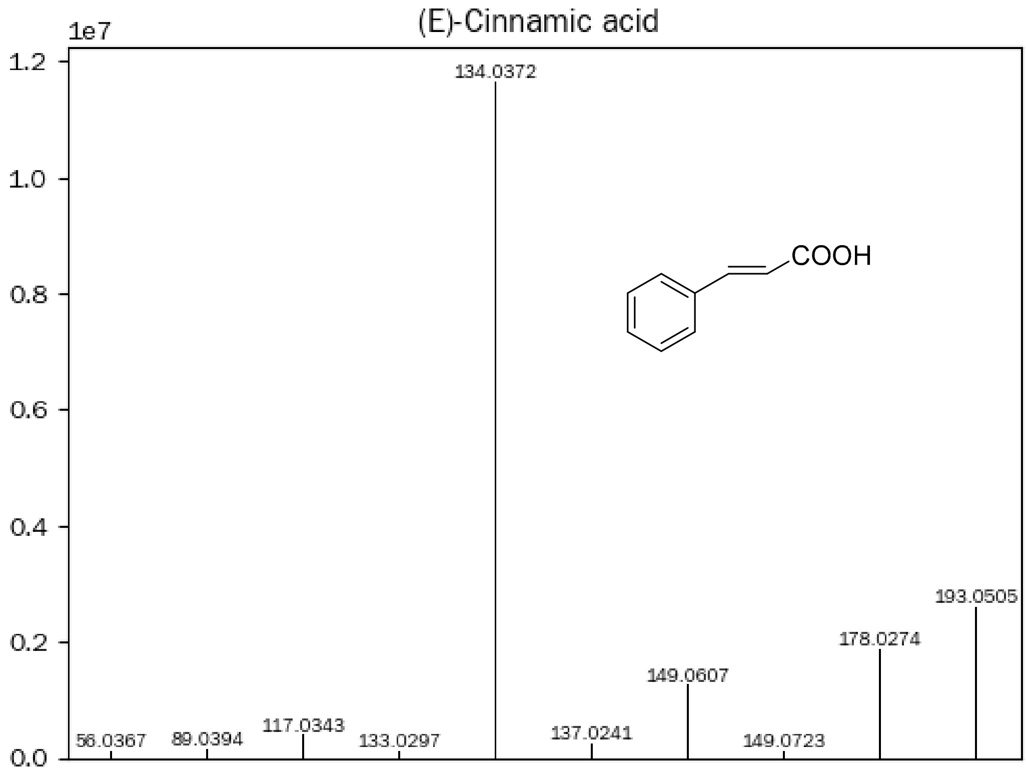

Figure S26. MS/MS2 spectrum of (E)-Cinnamic acid

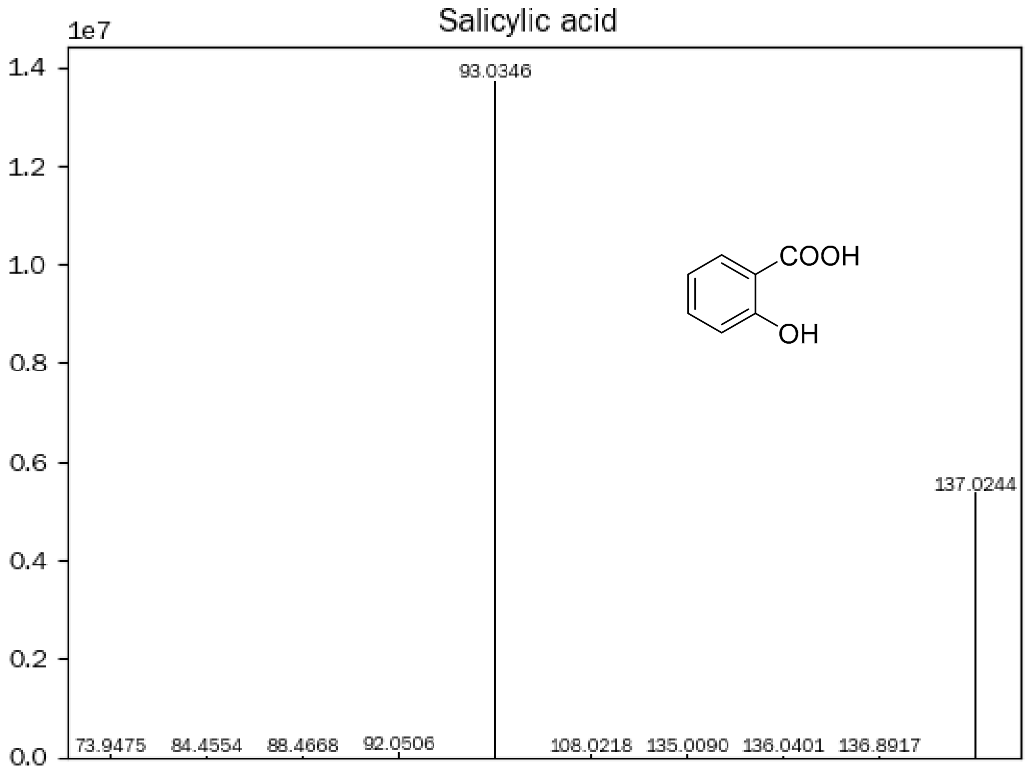

Figure S27. MS/MS2 spectrum of salicylic acid

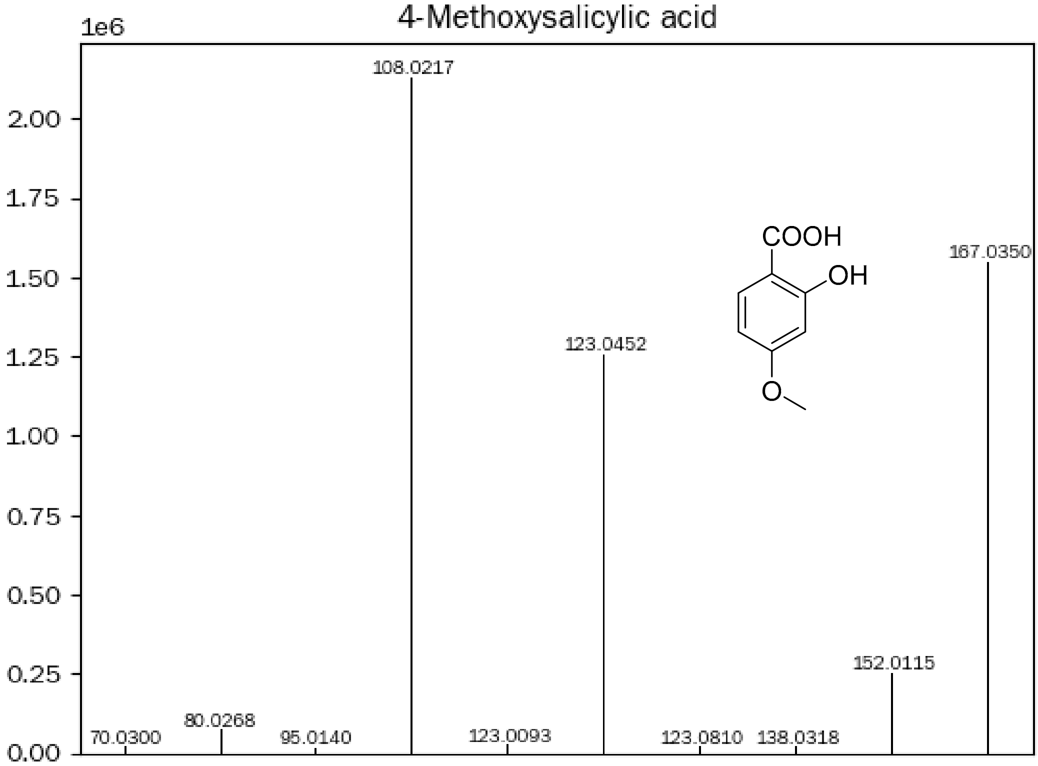

Figure S28. MS/MS2 spectrum of 4-Methoxysalicylic acid

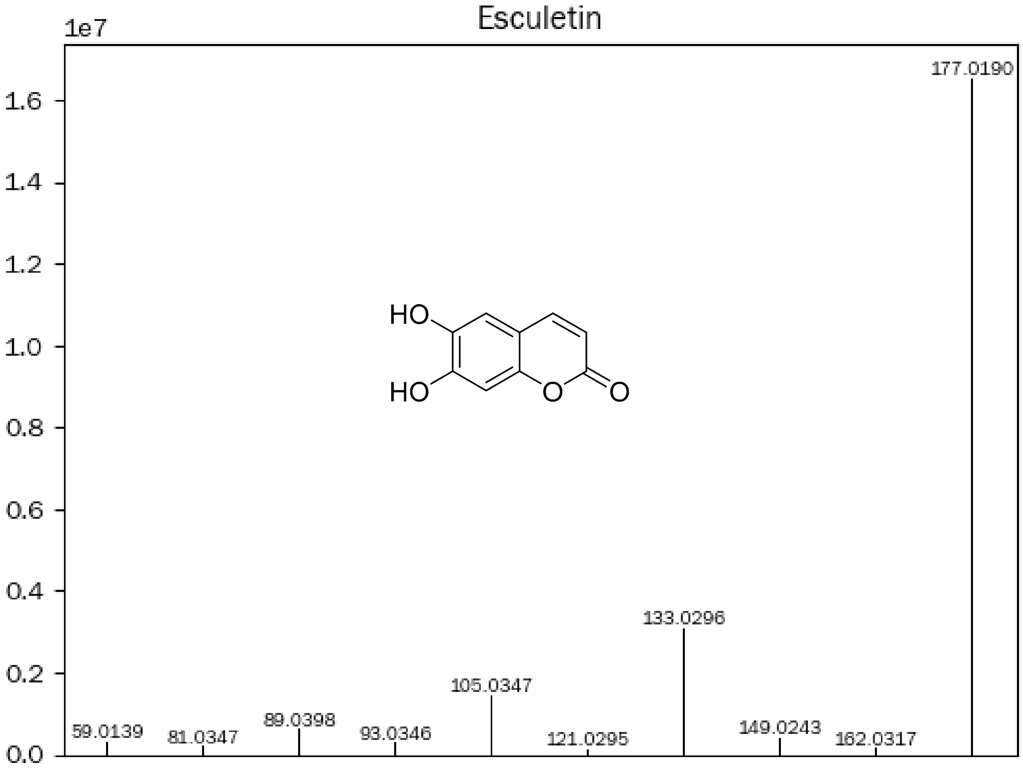

Figure S29. MS/MS2 spectrum of esculetin

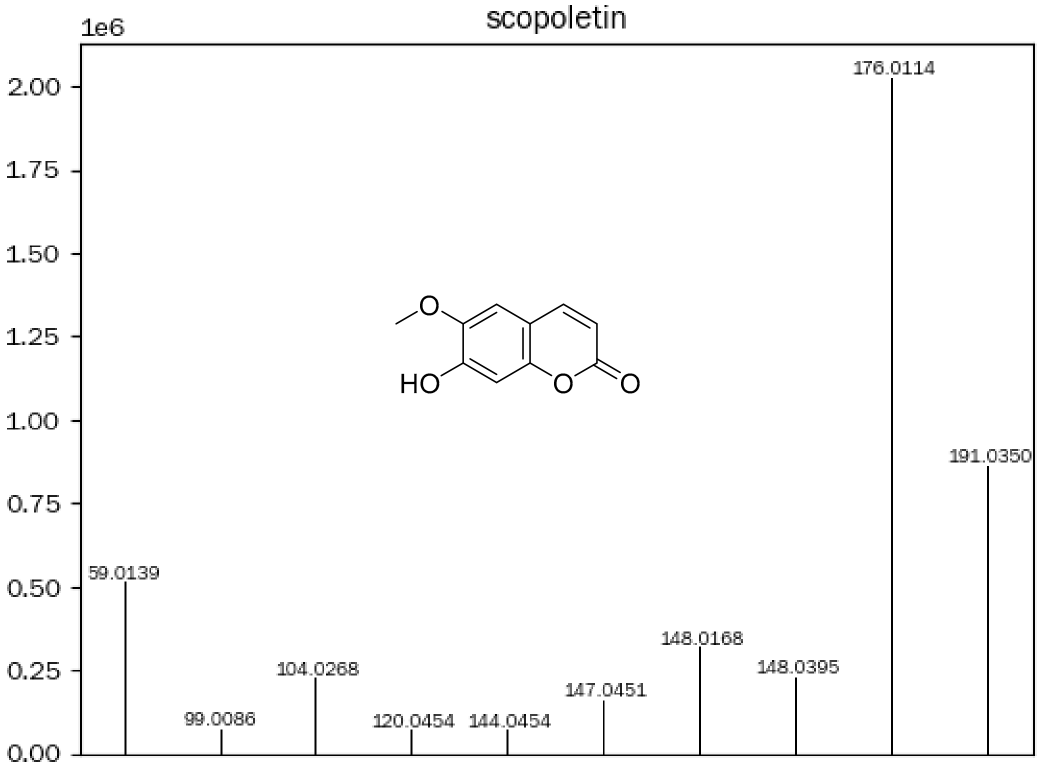

Figure S30. MS/MS2 spectrum of scopoletin

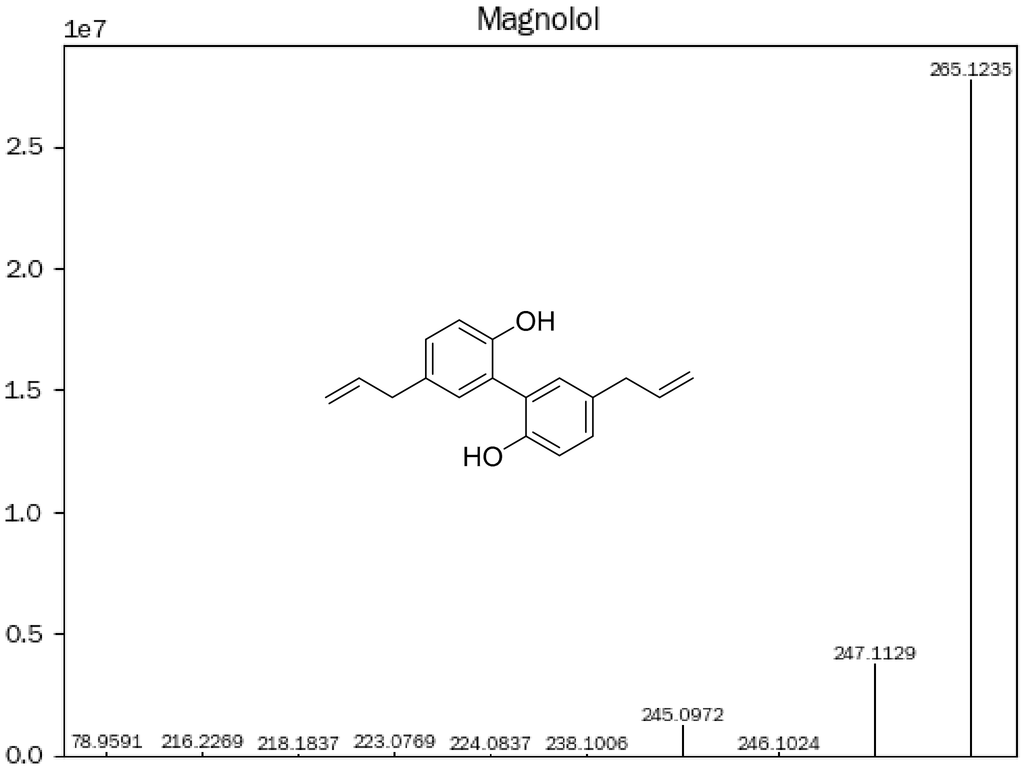

Figure S31. MS/MS2 spectrum of magnolol

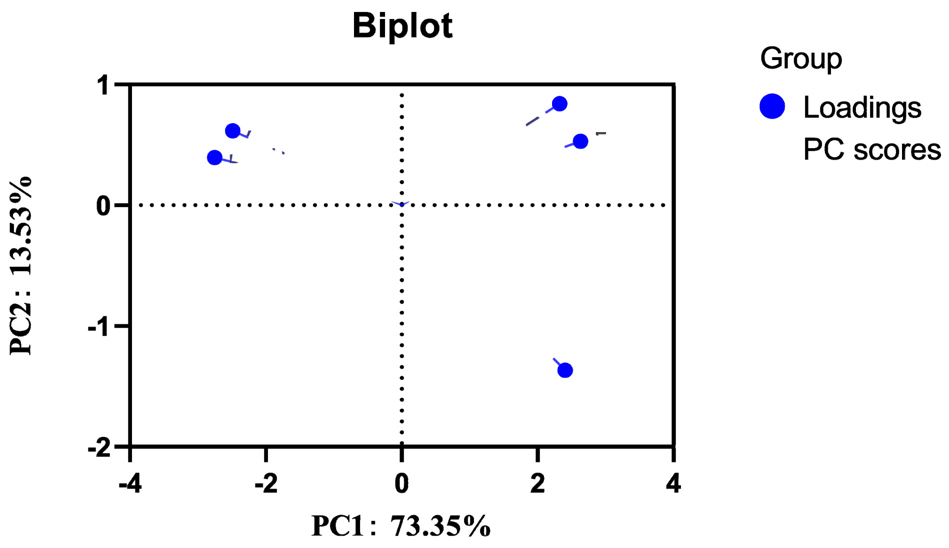

Figure S32. PCA scores plot showing the relationship among total flavonoid content (TFC), the total polyphenol content (TPC), and antioxidant activity (DPPH, ABTS, FRAP)

Principal component analysis (PCA) was conducted to obtain an overview of the correlation among total flavonoid content (TFC), total polyphenol content (TPC), and antioxidant activity (DPPH, ABTS, FRAP) (Figure S32). PCA results revealed that the first two principal components were able to explain 86.88% of the variability in the data. The first principal component (PC 1) described 73.35%, while the second (PC 2) was 13.53% of the total variance. Antioxidant activity (FRAP) was positively correlated with TFC and TPC, which exhibited acute angles with each other. It also suggested that the EtOAc fraction of peels from *P. erosus* exhibited the strongest Fe^{3+} reducing power (higher FRAP value) associated with higher content of TFC and TPC. DPPH and ABTS were located on the opposite side of TPC, this indicated that antioxidant activity (DPPH and ABTS) showed a negative correlation with TPC. Since DPPH and ABTS radical scavenging activity was reported in IC_{50}, the lower IC_{50} values indicated higher antioxidant activity, this negative correlation actually denoted their positive correlation. Hence, it indicated that the EtOAc fraction of peels from *P. erosus* showed the highest DPPH and ABTS radical scavenging activity related to the highest content of TPC.
